# High-Precision Determination of NMR Interaction Parameters by Measurement of Single Crystals: A Review of Classical and Advanced Methods

**DOI:** 10.3390/molecules29174148

**Published:** 2024-08-31

**Authors:** Thomas Bräuniger

**Affiliations:** Department of Chemistry, University of Munich (LMU), Butenandtstr. 5-13, 81377 Munich, Germany; thomas.braeuniger@cup.lmu.de; Tel.: +49-89-2180-77433

**Keywords:** single-crystal NMR spectroscopy, chemical shift, quadrupole interaction, tensor representation, goniometer probes

## Abstract

In this review, the process of extracting precise values for NMR interaction tensors from single crystal samples is systematically explored. Starting with a description of the orientation dependence of the considered interactions, i.e., chemical shift, dipolar, and quadrupole interaction, the techniques for acquiring and analysing single-crystal spectra are outlined. This includes the ‘classical’ approach, which requires the acquisition of three rotation patterns around three rotation axes that are orthogonal to each other, as well as more recent strategies aimed at reducing the number of required NMR spectra. One such strategy is the ‘single-rotation method’, which exploits the symmetry relations between tensors in the crystal structure to reduce the necessary amount of orientation-dependent data. This concept may be extended to additionally include the orientation of the goniometer axis itself in the data fit, which may be termed the ‘minimal-rotation method’. Other, more exotic schemes, such as the use of specialised probe designs or the investigation of single crystals under magic-angle-spinning, are also briefly discussed. Actual values of NMR interaction tensors as determined from the various single-crystal methods have been collected and are provided in tables for spin I=1/2, I=1, and half-integer spins with I>1/2.

## 1. Introduction

Since the early days of nuclear magnetic resonance (NMR) spectroscopy, it has been realised that performing measurements on single crystals allows us to retain some spectral resolution, which is easily lost for polycrystalline (powder) samples [1,2,3,4,5]. Apart from the fundamental Zeeman interaction for a nuclide with spin I≠0, the exact resonance frequency of an NMR line is determined by the electron density distribution around the observed nucleus (chemical shift, quadrupolar interaction) and sometimes also modified by the direct interaction of the magnetic momenta of the atoms (dipolar interaction). Due to the three-dimensional nature of these interactions, NMR resonance frequencies in solids are generally orientation-dependent, and therefore, usually described using second-rank tensors. In NMR solutions, usually only the isotropic parts of these tensors are observable because of averaging caused by rapid molecular motion. In solid-state NMR, it is possible to go beyond the isotropic parts and instead determine the full tensors. For polycrystalline samples, the principal tensor components may be identified from static spectra. However, this becomes problematic if, for the observed nuclide, several sites exist in the crystal structure (leading to spectral overlap) or if more than one sizeable interaction is present (e.g., both chemical shift and dipolar interactions), which may lead to complicated or obscure line shapes. But, even if the three principal tensor components may be deduced from static spectra, the spatial relation to the crystal structure is lost since all orientations are observed simultaneously in a powder sample. In contrast, from NMR of single crystals, the full tensors are accessible, meaning both their principal components (eigenvalues) and the orientation of their eigenvectors in the crystal lattice, which conveys information about the electron density distribution of the solid. The precision of tensor determination from single-crystal NMR may be so high that it is possible to detect subtle changes in the molecular shape induced by the crystal packing, as in a ^13^C-NMR study of naphthalene [6]. Other applications include the observation of phase transitions [7,8], the characterization of crystal twinning [9,10], and tracing isotopic disorder in the crystal lattice [11]. While far fewer papers are published on single-crystal NMR than on NMR studies of polycrystalline samples, the method is resorted to time and again in different contexts. Current progress in the field of single-crystal NMR was collected and commented on in a review by Vosegaard in 2021 [12].

The intention of the present article is a to provide a systematic description of how to acquire and process data to obtain the desired interaction tensors. For this, the ‘classical’ approach (necessitating three full rotations about three orthogonal axes), as well as more recent strategies aimed at reducing the amount of required data points (the ‘single-rotation method’), make use of standard hardware (instead of a dedicated goniometer set-up) by investigating single crystals with a magic-angle-spinning probe. Examples of actual NMR interaction tensors, which have been determined from various single-crystal methods, are provided in tables for spin I=1/2, I=1, and half-integer spins with I>1/2. However, before addressing these topics, the principles underlying NMR spectroscopy, in particular the various interactions leading to orientation dependence of the resonance signal, will be introduced systematically.

## 2. The NMR Resonance Line: The Origins of Orientation Dependence

Nuclear spin systems are quantum-mechanical entities, and as such, are described by appropriate Hamilton operators [13,14]. Since modern NMR spectrometers are mostly equipped with superconducting magnets, which create a comparatively strong magnetic flux density, evaluation of NMR spectra is usually carried out in the *high-field approximation* [15], where terms which do not commute with the Zeeman Hamiltonian are discarded. Solving the eigenvalue equation associated with the Hamilton operator of the spin system delivers energy eigenvalues, the differences between them, and the spectral transition energies. The resonance frequency of a given nuclide is a solid-state NMR experiment may be expressed as a sum of the various interactions contributing to the energy levels of a spin system:

(1) $$\nu \left(\Omega \right)=\nu_{0}+\nu_{\mathrm{CS}}\left(\Omega \right)+\nu_{\chi }\left(\Omega \right)+\nu_{\mathrm{DD}}\left(\Omega \right)\;\left(+\nu_{J}\left(\Omega \right)\right).$$

Here, $\nu_{0}$ stands for the Larmor frequency (the Zeeman interaction of the nuclear spin with the external magnetic field), $\nu_{\mathrm{CS}}$ for the chemical shift of the frequency induced by the shielding of the nucleus by the surrounding electrons, $\nu_{\chi }$ for the frequency change created by the interaction between the non-symmetric charge distribution of the nucleus (existing only for spins I>1/2) with the electronic surroundings, and $\nu_{\mathrm{DD}}$ for the frequency modification caused by through-space interaction of the nuclear magnetic dipoles. The $\nu_{J}$ term represents the effects of indirect *J*-couplings mediated via chemical bonds [16], which are mostly unobservable in solid-state NMR spectroscopy.

**Figure 1:**
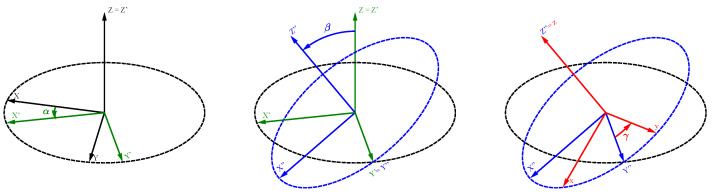
The Euler angles α, β, and γ, and the three Euler rotations, which carry the initial XYZ coordinate system into the final xyz coordinate system, according to the Rose convention [17].

The symbol Ω in Equation (1) is a general notation for the orientation dependence of the frequencies. This dependence could be expressed by a set of Euler angles [17,18], leading to Ω=(α,β,γ). These three angles allow the complete transformation from one coordinate system XYZ into a new system xyz by three consecutive rotations, as shown in Figure 1 (see Appendix A.1 for further details). It should be noted that the first two of these rotations, Ω=(α,β), correspond to the description of the orientation of a vector in the XYZ coordinate system by spherical coordinates, i.e., α may be identified with the azimuthal angle and β with the polar angle. Alternatively, orientation dependencies may be characterised by Wigner rotation matrices [19,20], or, as we will be doing in most of this paper, by a second-rank tensor representation [21,22].

### 2.1. The Chemical Shift

The chemical shift contribution $\nu_{\mathrm{CS}}\left(\Omega \right)$ is the small modification of the fundamental Larmor frequency $\nu_{0}$ caused by the shielding of the electron density surrounding the observed nucleus [23,24]. It is customary to report the observed frequency $\nu_{\mathrm{obs}}=\nu_{\mathrm{ref}}+\nu_{\mathrm{CS}}$ relative (and normalised) to a reference frequency $\nu_{\mathrm{ref}}$ as a numerical value δ without a unit, in parts per million (ppm):

(2) $$\delta =\frac{\nu_{\mathrm{obs}}-\nu_{\mathrm{ref}}}{\nu_{\mathrm{ref}}}\times 10^{6}.$$

The frequencies $\nu_{\mathrm{ref}}$ are measured from a range of established reference compounds [25], which have been chosen to be as close to the true Larmor frequency $\nu_{0}$ as possible. Using two Euler angles Ω=(α,β) to describe the orientation dependence, the frequency change caused by the chemical shift (in the high-field or secular approximation) may be written as follows:

(3) $$\nu_{\mathrm{CS}}\left(\alpha ,\beta \right)=\nu_{0}\delta_{\mathrm{iso}}+\nu_{0}\Delta \delta \left(\frac{3\cos^{2}\beta -1}{2}-\frac{\eta_{\mathrm{CS}}}{2}\sin^{2}\beta \cos2\alpha \right).$$

To understand the parameters employed in the above equation, we need to acknowledge that the proper mathematical expression to describe a three-dimensional electron distribution is a second-rank tensor. In an arbitrary coordinate system xyz, the chemical shift tensor $\delta^{xyz}$ can be expressed by a general 3×3 matrix. By an appropriate transformation Ω, the tensor $\delta^{xyz}$ can be transformed into its own principal axes system (PAS), where $\delta^{\mathrm{PAS}}$ has only its eigenvalues on the diagonal:

(4) $$\begin{array}{ccc} \delta^{xyz}=\left(\begin{array}{ccc} \delta_{xx} & \delta_{xy} & \delta_{xz}\\ \delta_{xy} & \delta_{yy} & \delta_{yz}\\ \delta_{xz} & \delta_{yz} & \delta_{zz} \end{array}\right),\; & \overset{\Omega }{⟷} & \delta^{\mathrm{PAS}}=\left(\begin{array}{ccc} \delta_{11} & 0 & 0\\ 0 & \delta_{22} & 0\\ 0 & 0 & \delta_{33} \end{array}\right). \end{array}$$

The scaled trace of the above tensors (which is invariant under transformations) is the isotropic chemical shift, the only parameter which is observable in NMR of solutions:

(5) $$\delta_{\mathrm{iso}}=\frac{1}{3}\sum_{i}\delta_{ii}.$$

We note that $\delta^{xyz}$ in Equation (4) is a symmetric tensor with only six independent components. This is not a fundamental property, but introduced by convention [26,27], because the asymmetric part of the full chemical shift tensor does not affect the line position. Taking the $\delta_{ii}$ components of the diagonalised chemical shift tensor $\delta^{\mathrm{PAS}}$, the following additional parameters may be defined:

(6) $$\eta_{\mathrm{CS}}=\frac{\delta_{22}-\delta_{11}}{\Delta \delta }\;\;\Delta \delta =\delta_{33}-\delta_{iso}.$$

Here, $\eta_{\mathrm{CS}}$ is the asymmetry parameter, which encapsulates information about the line shape of a polycrystalline sample (see below), and Δδ is the reduced anisotropy, which is a measure of the width of this line shape. For the definitions of Equation (6) to work properly, the eigenvalues $\delta_{ii}$ have to be sorted according to their absolute distance from $\delta_{\mathrm{iso}}$, by the so-called Haeberlen convention [15]:

(7) $$\left|\delta_{33}-\delta_{\mathrm{iso}}\right|\geq \left|\delta_{11}-\delta_{\mathrm{iso}}\right|\geq \left|\delta_{22}-\delta_{\mathrm{iso}}\right|.$$

**Figure 2:**
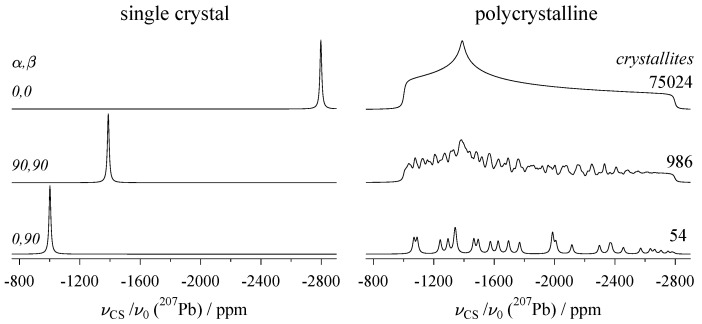
^207^Pb-NMR spectra (computer-generated with the Simpson package [32]) of the 6h site in vanadinite, with $\delta_{\mathrm{iso}}=-1729$ ppm, Δδ=−1071 ppm, and $\eta_{\mathrm{CS}}=0.36$, see Table A1 for $\delta_{ii}$ values. (**Left**): Single-crystal spectra at the indicated Euler angles α,β. (**Right**): Spectra of polycrystalline samples with the indicated number of crystallites distributed according to the ZCW scheme [33,34,35].

Thus, the parameters $\delta_{\mathrm{iso}}$, $\eta_{\mathrm{CS}}$, and Δδ used in Equation (3) as an alternative to the principal components $\delta_{11}$, $\delta_{22}$, and $\delta_{33}$ are now understood (other choices of alternative parameters exist, for example, the Herzfeld–Berger convention [28]). The angles Ω=(α,β) in the second term of this equation describe the orientation of the eigenvector $d_{33}$ associated with the largest eigenvalue (i.e., $\delta_{33}$) relative to the laboratory frame, where the vector $\vec{b}$ along the magnetic field lines defines the *z*-axis. The resonance positions resulting from three special values of α,β are shown on the left of Figure 2. From the top, $d_{33}$ is aligned along the *z*-, *y*-, and *x*-axis, respectively, projecting the eigenvalues $\delta_{33}$, $\delta_{22}$, and $\delta_{11}$. The numerical values for these tensor elements are actually those of the 6h site in the mineral vanadinite, which are listed Table A1. This table compiles chemical shift tensors of the nuclide ^207^Pb (spin I=1/2) derived from NMR experiments on naturally grown single crystals. For ^207^Pb, measuring NMR interaction parameters on a static sample turns out to have an additional advantage, as for many lead-containing compounds, the ^207^Pb chemical shift is strongly temperature-dependent [29]. This property is exploited for temperature calibration of NMR probes, usually with a sample of lead nitrate [30]. When using magic-angle spinning (see below, Section 3) to determine $\delta_{\mathrm{iso}}$, the measurement needs to be corrected for the effects of friction heating [31], whereas for static samples, such correction is obviously unnecessary.

As already mentioned, NMR measurements of solids are usually performed on polycrystalline samples, sometimes also referred to as powders. For such samples, the line shape is the sum of the contributions of the *N* crystallites present in the powder, all with their own specific (and more or less randomly distributed) orientation $\alpha_{i}$ and $\beta_{i}$. Powder spectra for incomplete crystallite ensembles (N=54 and 986, which are impossible or difficult to evaluate), and a smooth spectrum with N=75,024 (from which the principal components of the ^207^Pb chemical shift tensor may be read out) are plotted on the right of Figure 2, again for the 6h site of vanadinite. For the computation of these line shapes, the distribution of the $\alpha_{i},\beta_{i}$ values is no trivial task, the aim being to distribute them as uniformly as possible over the unit sphere [36]. A frequently employed method is the ZCW scheme, named after Zaremba [33], Conroy [34], and Wolfsberg [35].

**Figure 3:**
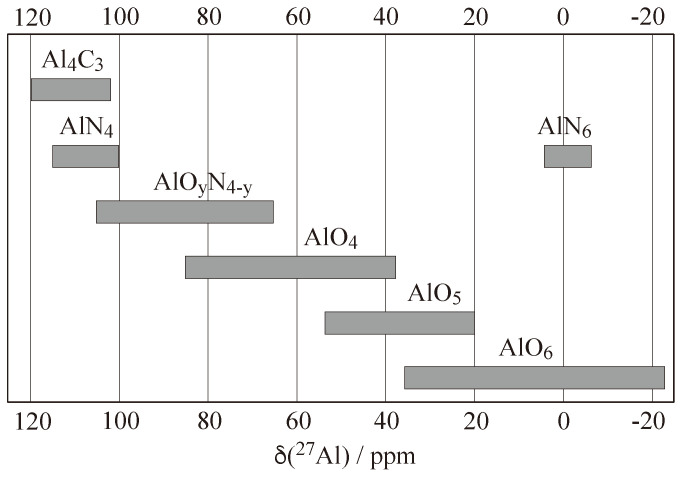
Ranges of isotropic chemical shift values for ^27^Al (I=5/2) for different oxygen/nitrogen coordination environments in periodic solids from the data provided in Table 1.

In general, chemical shift values are useful to the practising chemist because they can be correlated to structural features in the compounds under investigation, which is true not just for molecules [37], but also for periodic solids [38,39]. To illustrate this, the chemical shift ranges for the nuclide ^27^Al in various oxygen/nitrogen coordination environments are shown in Figure 3, with the actual numbers listed in Table 1.

Thus, recording a solid-state NMR spectrum of an aluminium-containing compound may aid the elucidation of the structure by identifying the coordination environment using the isotropic chemical shifts. There is, however, one *caveat* in the context of ^27^Al: since this nuclide possesses spin I=5/2, the quadrupolar interaction may also affect the position of the observed resonance line. This must be taken into account when determining the true $\delta_{\mathrm{iso}}$ values, as discussed below in Section 2.2.

### 2.2. The Quadrupole Interaction

The quadrupole interaction is an electrical interaction between the non-symmetric charge distribution of the nucleus (which is present for all nuclides with spin I>1/2) and the charge distribution of the electronic surroundings, described by the electric field gradient (EFG) tensor V. The elements $V_{ab}$ of the EFG tensor are defined as the second-order partial derivatives of the electric potential V(r), with *a* and *b* being any of the coordinates x,y,z [68]:

(8) $$V_{ab}=\frac{\partial^{2}V}{\partial q_{a}\partial q_{b}}{|}_{r=0}.$$

The product of the nuclear property eQ (with *Q* being the quadrupole moment of the nucleus [69]) and the largest eigenvalue $V_{33}$ of the EFG-tensor is called the quadrupolar coupling constant χ (sometimes also designated as $C_{Q}$ or $C_{q}$):

(9) $$\chi =C_{Q}=\frac{eQ}{h}V_{33}=\frac{eQeq}{h}.$$

The quadrupolar interaction $\nu_{\chi }$ is often treated as a perturbation to the main Zeeman interaction energy [19,70]. $\chi \ll \nu_{0}$, including only the first-order term of the perturbation, is usually sufficient, but for larger values of χ, terms of higher order may have to be taken into account as well to match the experimental findings:

(10) $$\nu_{\chi }\left(\Omega \right)=\nu_{\chi }^{\left(1\right)}+\nu_{\chi }^{\left(2\right)}+\nu_{\chi }^{\left(3\right)}+\ldots .$$

An alternative to this perturbation approach is direct calculations using matrix representations of either Hamiltonian or Liouvillian operators, which provide very precise results [71,72]. Nevertheless, the hierarchical concept provided by the perturbation method has its advantages in picturing the behaviour of the spin system. It is, for example, possible to separate the effects of the different perturbation orders by suitable combinations of the satellite frequencies, as shown below in Section 6.

To continue with parameter definitions, the ‘quadrupolar frequency’ $\nu_{Q}$, introduced by Cohen and Reif already in 1957 [68], is a useful measure of the interaction strength:

(11) $$\nu_{Q}=\frac{3e^{2}qQ}{2I(2I-1)h}=\frac{3\chi }{2I(2I-1)}.$$

Also, to describe the quadrupolar contributions to ν(Ω) in Equation (1), it is helpful to assign a parameter *k* to each transition in the spin system:

(12) $$k=m\pm \frac{1}{2}\;\mathrm{for}\;|m\rangle \to |m\pm 1\rangle .$$

With the above definitions, we can now proceed to specify the individual frequencies $\nu_{\chi }^{\left(i\right)}$ in Equation (10). The first-order contribution is given as follows:

(13) $$\nu_{\chi }^{\left(1\right)}\left(k\right)=k\frac{\nu_{Q}}{2}\left(3\cos^{2}\beta -1+\eta_{Q}\cos2\alpha \sin^{2}\beta \right).$$

Similar to the chemical shift, the angles Ω=(α,β) relate to the orientation of the eigenvector associated with the largest eigenvalue of the quadrupole coupling tensor Q in the laboratory frame. This Q tensor may be expressed in an arbitrary coordinate system xyz or in diagonal form in its PAS, with Ω defining the necessary transformation:

(14) $$\begin{array}{ccc} Q^{xyz}=\left(\begin{array}{ccc} Q_{xx} & Q_{xy} & Q_{xz}\\ Q_{xy} & Q_{yy} & Q_{yz}\\ Q_{xz} & Q_{yz} & Q_{zz} \end{array}\right),\; & \overset{\Omega }{⟷} & Q^{\mathrm{PAS}}=\left(\begin{array}{ccc} Q_{11} & 0 & 0\\ 0 & Q_{22} & 0\\ 0 & 0 & Q_{33} \end{array}\right). \end{array}$$

The $\eta_{Q}$ in Equation (13) is the quadrupolar asymmetry parameter:

(15) $$\eta_{Q}=\frac{Q_{11}-Q_{22}}{Q_{33}},$$

where the eigenvalues of $Q^{\mathrm{PAS}}$ need to be ordered according to the following:

(16) $$|Q_{33}\left|\geq \right|Q_{22}\left|\geq \right|Q_{11}|.$$

The Q tensor in its PAS can be reconstructed from the quadrupole coupling constant χ and the asymmetry parameter $\eta_{Q}$ as follows:

(17) $$Q^{\mathrm{PAS}}=\chi \left(\begin{array}{ccc} -\frac{1}{2}\left(1-\eta_{Q}\right) & 0 & 0\\ 0 & -\frac{1}{2}\left(1+\eta_{Q}\right) & 0\\ 0 & 0 & 1 \end{array}\right).$$

The quadrupole coupling tensor is generally traceless, $Q_{11}+Q_{22}+Q_{33}=0$. It is also symmetric, but in contrast to the chemical shift tensor δ, this is an intrinsic property following from the mixed partial derivatives in the definition of the EFG tensor V, see Equation (8), to which the quadrupole coupling tensor is connected by the following:

(18) $$Q=\frac{eQ}{h}V.$$

For stronger quadrupolar coupling (where ‘strong’ is always defined by the magnitude of the coupling $\nu_{Q}$, in relation to the Larmor frequency $\nu_{0}$), the second-order contribution $\nu^{\left(2\right)}$ needs to be included in the frequency calculation. For a static sample, this may be written as follows:

(19) $$\nu_{\chi }^{\left(2\right)}\left(k^{2}\right)=-\frac{\nu_{Q}^{2}}{6\nu_{0}}\left[\left(I^{2}+I-\frac{3}{4}-3k^{2}\right)g\left(\alpha ,\beta ,\eta_{Q}\right)-6k^{2}f\left(\alpha ,\beta ,\eta_{Q}\right)\right].$$

Here, functions *g* and *f* have terms depending on $\cos^{4}\beta$ and $\cos^{2}\beta$, with the dependencies on α and $\eta_{Q}$ encapsulated in the coefficients $A^{\left(2\right)},B^{\left(2\right)},\ldots$, which are listed in Appendix C:

(20) $$\begin{array}{c} \begin{array}{cc} g(\alpha ,\beta ,\eta_{Q}) & =A^{\left(2\right)}\cos^{4}\beta +B^{\left(2\right)}\cos^{2}\beta +C^{\left(2\right)}\\ f(\alpha ,\beta ,\eta_{Q}) & =D^{\left(2\right)}\cos^{4}\beta +E^{\left(2\right)}\cos^{2}\beta +F^{\left(2\right)} \end{array}. \end{array}$$

For very strong quadrupolar coupling, effects of third order might have to be added to the calculations, which for the static case may be expressed by the following:

(21) $$\begin{array}{c} \begin{array}{cc} \nu_{\chi }^{\left(3\right)}\left(k,k^{3}\right)=-k\frac{\nu_{Q}^{3}}{12\nu_{0}^{2}} & \{\left[12I\left(I+1\right)-40k^{2}-17\right]u\left(\alpha ,\beta ,\eta_{Q}\right)\\ \; & -2\left[3I\left(I+1\right)-5k^{2}-\frac{19}{4}\right]v\left(\alpha ,\beta ,\eta_{Q}\right)\\ \; & -3\left[8I\left(I+1\right)-20k^{2}-10\right]w\left(\alpha ,\beta ,\eta_{Q}\right)\} \end{array}. \end{array}$$

The functions u,v,w now depend on $\cos^{n}\beta$ with n=6,4,2. Again, the dependencies on α and $\eta_{Q}$ are encapsulated in the coefficients $A^{\left(3\right)},B^{\left(3\right)},\ldots$, and also given in Appendix C:

(22) $$\begin{array}{c} \begin{array}{cc} u(\alpha ,\beta ,\eta_{Q}) & =A^{\left(3\right)}\cos^{6}\beta +B^{\left(3\right)}\cos^{4}\beta +C^{\left(3\right)}\cos^{2}\beta +D^{\left(3\right)}\\ v(\alpha ,\beta ,\eta_{Q}) & =E^{\left(3\right)}\cos^{6}\beta +F^{\left(3\right)}\cos^{4}\beta +G^{\left(3\right)}\cos^{2}\beta +H^{\left(3\right)}\\ w(\alpha ,\beta ,\eta_{Q}) & =I^{\left(3\right)}\cos^{6}\beta +J^{\left(3\right)}\cos^{4}\beta +K^{\left(3\right)}\cos^{2}\beta +L^{\left(3\right)} \end{array}. \end{array}$$

#### 2.2.1. Quadrupole Interaction for Half-Integer Spin I>1/2

For nuclides with half-integer spin I>1/2, the quadrupole interaction separates the 2I observable resonances into the central transition (CT), for which the parameter *k* (Equation (12)) is zero, and (2I−1) satellite transitions (ST’s) with k=±1,±2,… Here, one advantage of using *k* instead of *m* becomes apparent: for a given transition, the sign of *k* does not depend on the direction in which this transition is traversed. Using the −5/2 satellite as an example, which could be considered either as |−5/2〉→|−3/2〉 or |−3/2〉→|−5/2〉 transition, it can be seen that for both cases, k=(−5+1)/2=(−3−1)/2=−2.

**Figure 4:**
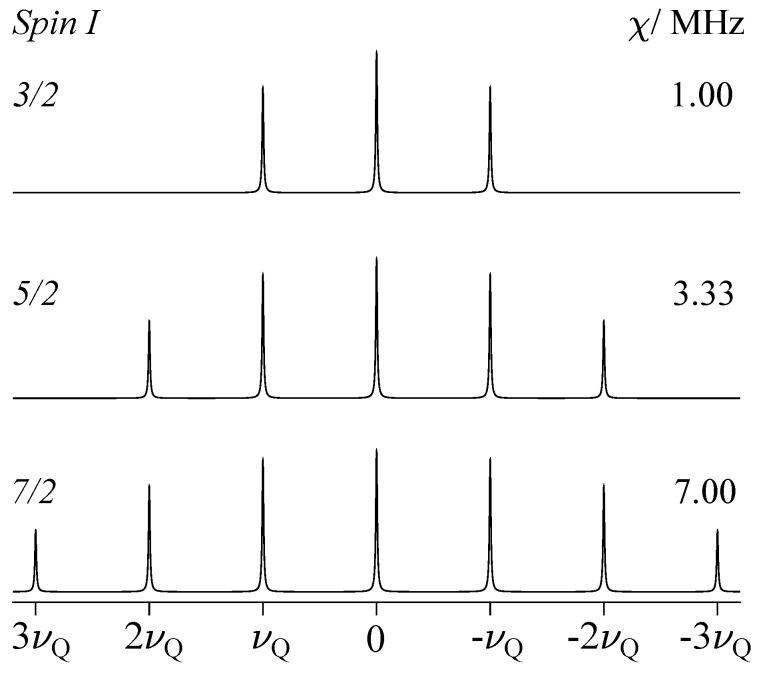
Single-crystal NMR spectra (computer-generated [32]) of nuclides with half-integer spin and $\nu_{Q}=0.5\;\mathrm{MHz}$, showing the maximum satellite displacement for a uniaxial Q tensor (i.e., $\eta_{Q}=0$) at $\beta_{\max}=0$, see Equation (23). To keep $\nu_{Q}$ constant, the respective quadrupole coupling constants χ need to be adjusted as indicated on the right.

For comparatively weak quadrupole coupling, i.e., for small values of $\nu_{Q}$, it is sufficient to take the quadrupole interaction into account to first-order only; that is, $\nu_{\chi }\left(\Omega \right)=\nu_{\chi }^{\left(1\right)}$. Thus, the STs are shifted away from the CT and form symmetrical doublets around the central transition, whereas for the CT with k=0, no shift occurs, as shown schematically for spins I=3/2, 5/2, and 7/2 in Figure 4. For a uniaxial Q tensor (with $Q_{11}=Q_{22}$, and therefore $\eta_{Q}=0$), Equation (13) reduces as follows:

(23) $$\nu_{\chi }^{\left(1\right)}=k\frac{\nu_{Q}}{2}\left(3\cos^{2}\beta -1\right).$$

The orientation-dependent term of $\nu_{\chi }^{\left(1\right)}$ has its maximum value for $\beta_{\max}=0$; hence, the maximum displacement experienced by the satellite transitions is as follows:

(24) $$\nu_{\chi }^{\left(1\right)}\left(\beta_{\max}\right)=k\nu_{Q}\;\;\mathrm{with}\;\nu_{Q}=\frac{3\chi }{2I(2I-1)}\;\;\mathrm{and}\;k=\pm 1,\pm 2,\ldots$$

**Figure 5:**
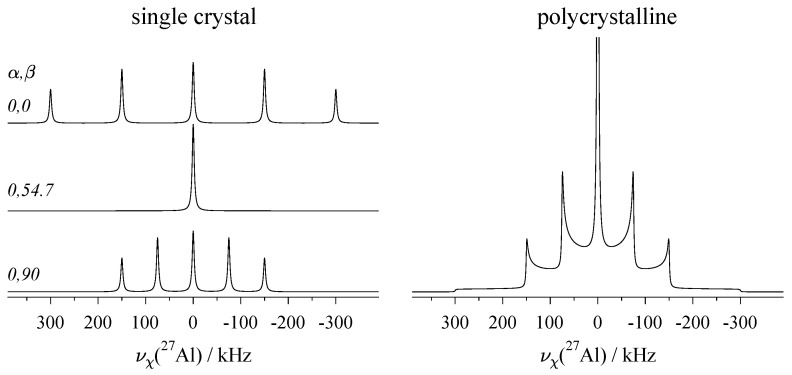
^27^Al-NMR spectra (computer-generated [32]) of a ^27^Al nucleus (spin I=5/2) with χ=1.0 MHz and $\eta_{Q}=0$, under quadrupole interaction to first order, see Equation (13). (**Left**): Single-crystal spectra at the indicated Euler angles α,β. (**Right**): Spectrum of a static polycrystalline sample, with the distinct satellite singularities corresponding to $\beta =90^{\circ }$. The very intense central-transition peak has been cut off to improve the visibility of the satellite pattern.

Under first-order, for the satellites of any nuclide with half-integer spin I>1/2 and $\eta_{Q}=0$, the ±k resonances at maximum shift are spaced by the quadrupolar frequency $\nu_{Q}$, as shown in Figure 4. Here, the numerical value of $\nu_{Q}$ is kept constant at a value of 0.5 MHz, necessitating an adjustment of the magnitude of the quadrupolar coupling constant χ, as indicated in the graphics. The satellite positions may slightly deviate from this $\nu_{Q}$ convention for biaxial tensors (with $Q_{11}\neq Q_{22}$) and under higher-order contributions of the quadrupole interaction, as discussed below. Figure 5 shows examples of the general orientation dependence of $\nu_{\chi }^{\left(1\right)}$, using ^27^Al with spin I=5/2 as the model system. Single-crystal spectra for some combinations of Euler angles α,β are shown on the left, and the static spectrum of a polycrystalline sample, where all orientations are present simultaneously, is shown on the right.

Under quadrupole interaction to second order, which is needed for larger values of $\nu_{Q}$, the frequency contribution is given by $\nu_{\chi }\left(\Omega \right)=\nu_{\chi }^{\left(1\right)}+\nu_{\chi }^{\left(2\right)}$. The two resonances of a satellite transition doublet belonging to ±k are being shifted in the same direction by $\nu_{\chi }^{\left(2\right)}$, irrespective of the sign of *k*. This is easy to see in Equation (19), since the parameter only shows up in the form of $k^{2}$. It also means that under second order, the two resonances of the ST doublet are no longer placed symmetrically around the central transition. In fact, both STs and CTs may move, and in contrast to the first-order contribution, the CT resonance with $k^{2}=0$ is now also affected by the quadrupole interaction. Its position shifts according to a shortened version of Equation (19):

(25) $$\nu_{\chi }^{\left(2\right)}\left(k^{2}=0\right)=-\frac{\nu_{Q}^{2}}{6\nu_{0}}\left(I^{2}+I-\frac{3}{4}\right)g\left(\alpha ,\beta ,\eta_{Q}\right).$$

**Figure 6:**
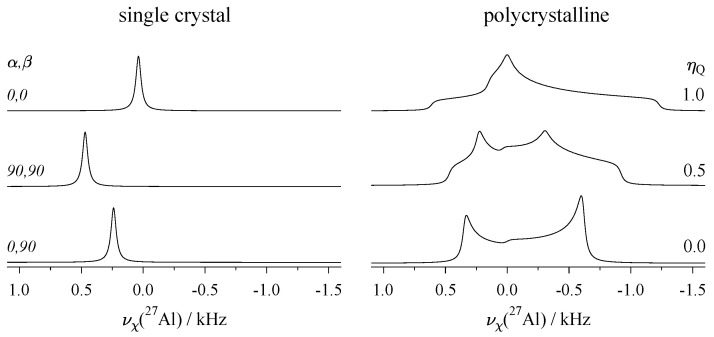
^27^Al-NMR central-transition spectra (computer generated [32]) of a ^27^Al nucleus (I=5/2) with $\nu_{0}=130.3$ MHz, χ=2.0 MHz and the indicated $\eta_{Q}$ parameters under quadrupole interaction to second-order, see Equation (25). (**Left**): Single-crystal spectra for $\eta_{Q}=0.5$ at the indicated Euler angles α,β. (**Right**): Spectra of a static polycrystalline sample for $\eta_{Q}=0.0,0.5$ and 1.0. The scaling of the frequency axis differs from that used in Figure 5 by two orders of magnitude.

In Figure 6, both single-crystal and powder spectra of the central transition under the second-order quadrupole effects are shown. Since these effects are scaled down by the Larmor frequency $\nu_{0}$, they are much smaller than the first-order displacement experienced by the STs pictured in Figure 5, and may be reduced or even completely suppressed by higher magnetic field strengths.

Fpr the third order, the contribution is described by $\nu_{\chi }\left(\Omega \right)=\nu_{\chi }^{\left(1\right)}+\nu_{\chi }^{\left(2\right)}+\nu_{\chi }^{\left(3\right)}$. The two resonances of the satellite pair belonging to ±k are shifted in opposite directions by $\nu_{\chi }^{\left(3\right)}$ (with Equation (21) depending on both *k* and $k^{3}$), whereas the central transition with k=0 is not affected at all [73]. The third-order effect on the resonance positions is thus similar to that of the first-order contribution, but, being scaled by $\nu_{0}^{-2}$, is of a much smaller magnitude.

By suitable combinations of resonance frequencies, it is possible to selectively remove some contributions while retaining others. This permits the determination of the full Q tensor from rotation-dependent single crystal data, as detailed below. Some examples of quadrupole coupling tensors derived from single-crystal NMR experiments for various nuclides with half-integer spin I>1/2 are provided in Table A2.

#### 2.2.2. Quadrupole Interaction for Integer Spin I=1

**Figure 7:**
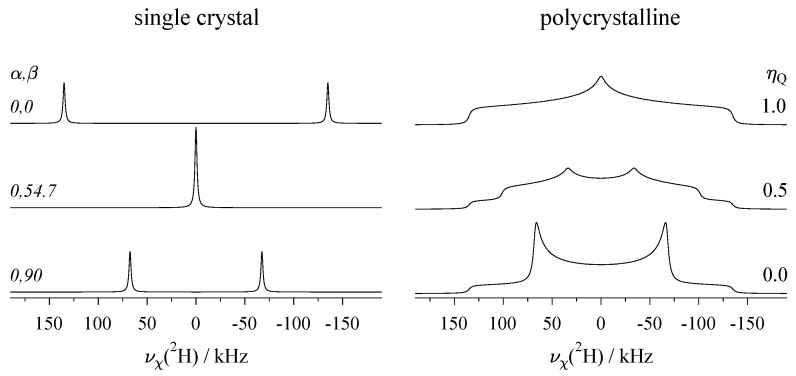
^2^H-NMR spectra (computer-generated [32]) of a deuterium nucleus (spin I=1) with χ=180 kHz, under quadrupole interaction to first-order, see Equation (26). (**Left**): Single-crystal spectra for $\eta_{Q}=0$ at the indicated Euler angles α,β. (**Right**): Spectra of a static polycrystalline sample, for $\eta_{Q}=0.0,0.5$ and 1.0.

For quadrupole nuclides with integer spin, the case I=1 has by far the most applications, as it includes the isotopes ^2^H [74,75] and ^14^N [76]. For spin-1 nuclei, three energy levels with m=−1,0,+1 and two transitions exist. These two transitions are described by k=±1/2 (see Equation (12)), and to first-order, show up as a doublet with two components $\nu_{+}^{\left(1\right)}$ and $\nu_{-}^{\left(1\right)}$ in the single-crystal spectrum, as shown in Figure 7. The general equation for the quadrupole frequency to first-order (Equation (13)) changes for spin I=1 to the following form:

(26) $$\nu_{\pm }^{\left(1\right)}=\pm \frac{\nu_{Q}}{4}\left(3\cos^{2}\beta -1+\eta_{Q}\cos2\alpha \sin^{2}\beta \right).$$

In Figure 7, single-crystal and powder spectra of deuterium (^2^H) are displayed. Because of the structure of the orientation-dependent term, the largest displacement of the resonances occurs for $\beta =0^{\circ }$, and about half that displacement for $\beta =90^{\circ }$ (the exact factor of −1/2 applies only for $\eta_{Q}=0$), with the resonances of the I=1 doublet having changed sides. In between, at the angle $\beta_{0}$ (see also below, Section 3), the first-order quadrupolar shift is zero, and the two resonances of the doublet coincide. The same principle applies to the spectra of nuclides with half-integer spin I>1/2, as depicted above in Figure 5.

For deuterium atoms bound to other atoms with a covalent single bond, the electron distribution around this bond tends to be cylindrically symmetric. This leads to the following rules about the quadrupole coupling tensor Q for deuterium, as formulated by Haeberlen in 2001 [77]:

(i) The Q tensors of a deuterium atom in a covalent bond are nearly axially symmetric, with the asymmetry parameter remaining in the range of $0\leq \eta_{Q}\leq 0.1$.
(ii) The direction of the eigenvector associated with the largest principal component of the Q tensor ($Q_{33}$) is parallel to the bond direction of the deuteron.
(iii) If the deuteron is part of a planar structure (e.g., aromatic rings), the eigenvector of the second-largest principal component ($Q_{22}$) is perpendicular to this plane.

Interestingly, in the same work [77], it was shown by combining a high-precision single-crystal NMR study of α-Ca(DCOO)_2_ with X-ray and neutron diffraction results, that small deviations from the above rules (ii) and (iii) may occur. From the fact that $\eta_{Q}$ of covalently bound deuterium atoms is always small, it follows that spectra with pronounced asymmetry (such as those shown on the right of Figure 7 with $\eta_{Q}=0.5$ and 1.0) show up only in systems where dynamic processes have created an averaged Q tensor with reduced magnitude [7,78,79,80]. Table A3 lists examples of quadrupole coupling parameters for deuterium in organic, aromatic compounds, which are static on the NMR time scale. The ^2^H nucleus carries a comparatively small quadrupole moment of Q=2.86 mb [69], and the corresponding quadrupole coupling constants χ found for deuterium thus do not exceed 200 kHz. Therefore, treating the quadrupole interaction to first-order (Equation (26)) is sufficient in very good approximation. The situation is much different for ^14^N, which has a much larger quadrupole moment of Q=20.44 mb [69], leading to strong effects of the quadrupole interaction on the NMR spectra. Direct acquisition of ^14^N is usually only possible for nitrogen in highly symmetric structures [66,81,82,83,84], with all other cases requiring specialised methods such as wide-line or overtone spectroscopy [76].

### 2.3. The Dipolar Interaction

The dipolar interaction describes the energetic interplay of the nuclear magnetic moments with each other. In solution NMR (or in solids undergoing dynamic processes on the appropriate time scale), it is motionally averaged out and does not have any effects on the resonance position. In rigid solids, however, the magnetic dipoles ‘see’ each other long enough to have an effect on the energy levels of the spin system. The quite extensive Hamiltonians describing this dipolar energy can be truncated in the high-field approximation, leaving two fundamental cases: interaction between identical types of nuclides (homonuclear case) and between different types (heteronuclear case). For an isolated spin pair *i* and *j*, the dipolar interaction splits the resonance into two components $\nu_{+}$ and $\nu_{-}$, with the frequencies of the doublet described as follows:

(27) $$\begin{array}{ccc} \nu_{\pm }^{\mathrm{hetero}} & = & \pm \frac{1}{2}b_{ij}\left(3\cos^{2}\theta -1\right)\\ \nu_{\pm }^{\mathrm{homo}} & = & \pm \frac{3}{4}b_{ij}\left(3\cos^{2}\theta -1\right) \end{array}.$$

Here, θ is the angle between the internuclear vector and the external magnetic field. The dipolar coupling constant $b_{ij}$ contains the gyromagnetic ratios γ of the nuclei and scales with the inverse cube of their distance:

(28) $$b_{ij}=-\frac{\mu_{0}}{8\pi^{2}}\frac{\gamma_{i}\gamma_{j}\hbar }{r_{ij}^{3}}.$$

According to Equation (27), the largest splittings $\Delta \nu =(\nu_{+}-\nu_{-})$ (being $2b_{ij}$ for the heteronuclear and $3b_{ij}$ for the homonuclear case) are observed when the internuclear vector is oriented parallel to the external magnetic field, i.e., θ=0. If determination of $b_{ij}$ is feasible from the spectrum, information about the distance $r_{ij}$ between the coupling spins is available via Equation (28). Polycrystalline (powder) spectra dominated by the dipolar interaction were first described by Pake in 1948 [85], and are sometimes referred to as ‘Pake patterns’ in the literature.

The dipolar interaction will not be discussed further here, as in the context of single-crystal NMR, it mostly leads to some unspecific line broadening of the resonances. However, if the couplings are well resolved, the dipolar coupling tensors may be determined. For a network of coupled spins, the coupling pattern tends to become very complex, see, for example, the ^31^P-NMR study of NH_4_H_2_PO_4_ by Eichele and Wasylishen [86]. Information about dipolar couplings may also be obtained from static, polycrystalline samples [85,87]. Again, this works well for an isolated spin pair but becomes increasingly difficult as more spins become involved in the coupling network. For such cases, it can be helpful to suppress the effects of dipolar couplings by application of spin decoupling, i.e., by irradiation of spin *i* while observing spin *j* [88,89,90].

### 2.4. Effect of the Various Interactions on Single-Crystal Spectra

For all NMR interactions discussed in this Section, the effects on the position of the resonance line are summarised in Table 2. In particular, the behaviour of chemical shift and quadrupolar interaction will be essential when evaluating single-crystal data to extract the full interaction tensors, as the varying signs of the contributions can be exploited to remove some interactions while keeping others. These procedures will be explained in detail in Section 6. A brief description of the nature of ‘cross terms’ (listed in the last column of Table 2) will be provided below in Section 3.

## 3. Averaging the Orientation Dependence: Magic-Angle-Spinning (MAS)

### 3.1. MAS of Polycrystalline Samples

As discussed above, the orientation dependence of various NMR interactions causes severe broadening of the resonance lines of polycrystalline samples. The form of this orientation dependence is identical to the chemical shift (Equation (3)), dipolar interaction (Equation (27)), and quadrupolar interaction of first-order (Equations (13) and (26)), namely the Legendre polynomial $P_{2}\left(\cos\beta \right)$:

(29) $$P_{2}\left(\cos\beta \right)=\frac{1}{2}\left(3\cos^{2}\beta -1\right)\;\beta_{0}=\arccos\left(\sqrt{1/3}\right).$$

While the shape of the broad powder spectra (as shown in Figure 2 and Figure 5, Figure 6 and Figure 7) may be analysed to extract the principal tensor components, the overlap of these line shapes (or the simultaneous presence of several interactions) can make such analysis difficult or even impossible. To regain resolution, the *magic-angle spinning* (MAS) technique may be employed [91]. For this, the sample is tightly packed into a rotor and spun around an axis tilted by $\beta_{0}$ (which is the root of $P_{2}$ in Equation (29)) against the direction of the magnetic field $B_{0}$. If the spinning is fast enough, the anisotropic interactions may be averaged out; hence, $\beta_{0}$ is also referred to as the *magic angle*.

**Figure 8:**
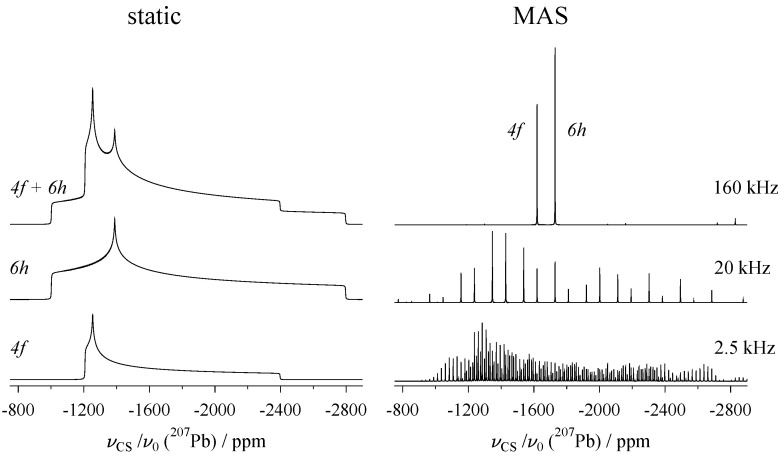
^207^Pb-NMR spectra of a polycrystalline sample of vanadinite (computer-generated [32]). (**Left**): Static powder spectra of the two distinct crystallographic sites 4f und 6h and their superposition (top). (**Right**): MAS spectra of (4f+6h) at the indicated rotation frequencies, with 2.5 and 20 kHz producing wide spinning sideband (SSB) patterns. At the (currently not yet realisable) MAS rate of $\nu_{rot}=160$ kHz, only the isotropic peaks, with the expected intensity ratio of 4:6, are still observable.

For a polycrystalline sample of nuclides, which are subjected only to chemical shift anisotropy, sufficiently fast spinning leaves a single narrow line at $\delta_{\mathrm{iso}}$, see Figure 8. Here, ‘sufficiently fast’ is defined by the ratio of rotation frequency $\nu_{\mathrm{rot}}$ to the frequency $\nu_{\mathrm{aniso}}$ of the interaction that needs to be averaged, i.e., in the range of kHz for the chemical shift, but in the range of MHz for the quadrupolar interaction. Typically, $\nu_{\mathrm{rot}}$ needs to be larger than $\nu_{\mathrm{aniso}}$ by a factor of 2 to 5 for full averaging [92]. In the intermediate regime, where a manifold of spinning side bands (SSBs) are still visible, the principal tensor components may be determined from the intensity distribution of the SSB pattern [28]. Because the SSBs focus the spectral intensities in comparatively few narrow lines (instead of having it dispersed over the full range of $\nu_{\mathrm{aniso}}$), derivation of tensor components from a polycrystalline sample is generally more reliable under MAS than under static conditions [93].

As may be seen from Equations (19) and (20), the expressions for the quadrupolar effects of second-order contain terms that scale with $\cos^{4}\beta$; that is, a Legendre polynomial of the fourth order. The same is true for the so-called cross terms between chemical shift and either dipolar [94,95] or quadrupolar interaction [96], where the coupling of two second-rank tensors leads to a tensor of the fourth rank. The effects of terms scaling with $\cos^{4}\beta$ on the NMR spectra cannot be averaged out by MAS; therefore, some residual line broadening will be present. This is particularly evident for the second-order broadened line shapes of the central transition under MAS conditions. To fully remove these effects, additional techniques need to be applied, e.g., double rotation for second-order quadrupolar effects [97] or spin decoupling by RF irradiation for cross terms [88,89,90].

### 3.2. MAS of Single Crystals

It has also been suggested to apply the magic-angle spinning method to single crystals [98,99,100]. In this approach, a small number of single crystals is placed in an MAS rotor and spun at a moderate frequency. Then, rotor-synchronized radiofrequency pulses are used, which generate a mixture of absorptive and dispersive line shape contributions caused by the different phases of crystallites with different orientations. By changing the timing of the pulses with respect to the rotor position, information about both the tensor eigenvalues and the tensor orientation (which cannot derived from a polycrystalline sample containing all possible orientations simultaneously) can be obtained. According to Jäger and co-workers [98], this method has the following advantages: (a) it provides better resolution than single-crystal spectra if dipolar couplings are present in the system (see Section 2.3), because the broadening caused by these couplings is averaged out by MAS, which, in turn, leads to improved signal-to-noise ratios and concomitant time savings; (b) it relies on standard MAS hardware, which obviates the need for a dedicated goniometer probe. In addition, the application of MAS may also be helpful for averaging anisotropies of the magnetic susceptibility [101], at least partly [102]. For static single crystals, susceptibility problems can be eliminated by shaping the crystal into a sphere [103,104]. Full chemical shift tensors derived from single crystals under MAS may be found in Table 3.

A variation of the above-described procedure is to use regular polycrystalline samples (and not just a few crystallites as in the method discussed above). Under MAS, selective RF pulses are applied to the full powder, exciting only 0.1–10% of the crystallites in the sample [105]. To some degree, this simplifies data analysis since the full integral over all orientations is reduced to a smaller subset. However, the absolute orientation of the tensors cannot be attained.

Finally, for MAS experiments, a single crystal may also be used as the sample container, as is the case for rotors made from single-crystalline α-Al_2_O_3_ (also known as sapphire or corundum). As an additional benefit, the ^27^Al-NMR spectrum of the rotor may be utilised to precisely set the magic angle [106].

## 4. Expressing the Orientation Dependence: The Tensor Representation

### 4.1. Deriving the Resonance Position from the Interaction Tensor

In Section 2, the orientation dependence of the various interaction frequencies ν(Ω) has been described using a set of two Euler angles, Ω=(α,β). These could also be understood as being the azimuthal and polar angles of the eigenvector associated with the largest eigenvalue of the respective tensor in the laboratory frame, where the magnetic field vector $\vec{b}$ defines the *z*-axis. This choice of orientation dependence was used in Equations (3), (13) and (26). For uniaxial tensors, only the polar angle β is sufficient to capture the orientation dependence, see Equations (23) and (27).

Instead of being concerned about the orientation of a tensor eigenvector, the interaction frequencies may also be computed from the tensor directly [107,108]. Generally, for an NMR interaction described by a second-rank tensor T, the corresponding NMR frequency $\nu_{T}$ in the high-field approximation may be obtained by the following vector–tensor–vector product, where $\vec{b}$ is the unit vector pointing along the lines of the external magnetic field, and ${\vec{b}}^{T}$ is the transposed vector (i.e., row instead of column):

(30) $$\nu_{T}={\vec{b}}^{T}\cdot T\cdot \vec{b}={\vec{b}}^{T}\cdot \left(\begin{array}{ccc} t_{xx} & t_{xy} & t_{xz}\\ t_{xy} & t_{yy} & t_{yz}\\ t_{xz} & t_{yz} & t_{zz} \end{array}\right)\cdot \vec{b}.$$

For the above equation to work, both $\vec{b}$ and T must be specified in the same coordinate system. The unit vector $\vec{b}$ may be expressed in either Cartesian or spherical coordinates:

(31) $$\vec{b}=\left(\begin{array}{c} x\\ y\\ z \end{array}\right)=\left(\begin{array}{c} \sin\theta \cos\phi \\ \sin\theta \sin\phi \\ \cos\theta \end{array}\right).$$

As a concrete example, the chemical shift may be considered as follows:

(32) $$\frac{\nu_{\mathrm{CS}}}{\nu_{0}}={\vec{b}}^{T}\cdot \left(\begin{array}{ccc} \delta_{xx} & \delta_{xy} & \delta_{xz}\\ \delta_{xy} & \delta_{yy} & \delta_{yz}\\ \delta_{xz} & \delta_{yz} & \delta_{zz} \end{array}\right)\cdot \vec{b}.$$

The measurements are performed in the LAB frame, where the magnetic field vector points along the *z*-axis, such that $\vec{b}=\left(\begin{array}{ccc} 0 & 0 & 1 \end{array}\right)$; therefore, the observed chemical shift is determined only by the $\delta_{zz}$ component of the $\delta^{\mathrm{LAB}}$ tensor. For a general coordinate system abc, an appropriate expression for ${\vec{b}}^{abc}$ must be found and multiplied by the $\delta^{abc}$ tensor, as shown above. For example, it may be advantageous to determine the tensor in the CRY frame, which has a direct relation to the electron density distribution of the crystal structure. Obviously, the frequency calculated from Equation (32) must be identical to that computed from Equation (3) provided in Section 2.1, where the orientation is defined by the polar and azimuthal angle. The equivalence of these two expressions is demonstrated in Appendix D.

The principle of Equation (30) may also be applied to calculate the frequency contributions of the quadrupole interaction to first-order from the quadrupole coupling tensor Q (see Equations (14) and (17)). For the central transition (k=0) and the satellite transitions (k=±1,±2,…) of nuclides with half-integer spin I>1/2, the frequencies of the various transitions *k* may be computed from the following:

(33) $$\nu_{\chi }^{\left(1\right)}=k\frac{\nu_{Q}}{\chi }\;{\vec{b}}^{T}\cdot Q\cdot \vec{b}=\frac{3k}{2I(2I-1)}\;{\vec{b}}^{T}\cdot Q\cdot \vec{b}.$$

For NMR interactions which split the Zeeman resonance into a doublet, both the positive $\nu_{+}$ and the negative component $\nu_{-}$ have to be taken into account. Thus, for spin I=1 under quadrupole interaction to first-order, the two transitions are described by k=±1/2, and the frequencies (Equation (26)) can be derived from the following tensor notation:

(34) $$\nu_{\pm }^{\left(1\right)}=k\frac{\nu_{Q}}{\chi }\;{\vec{b}}^{T}\cdot Q\cdot \vec{b}\;\overset{I=1}{=}\;\pm \frac{3}{4}\;{\vec{b}}^{T}\cdot Q\cdot \vec{b}.$$

### 4.2. Equivalence of Interaction Tensors in the Crystal Structure

Because of crystal symmetry, only a limited number of interaction tensors exists in every periodic structure, generating a limited number of NMR lines. The number of distinct resonances in a single crystal spectrum is decided by the number of spins that are *not* magnetically equivalent in the crystal lattice (the formal criterion for magnetic equivalence being that the operator of the symmetry element generating the second spin from the first must commute with the NMR–Hamiltonian of the spin under consideration):

**Crystallograpic Equivalence:**  Spins are connected by any symmetry element.
**Magnetic Equivalence:**  Spins are connected by either inversion or translation.

Thus, NMR interaction tensors of a given nuclide at a single crystallographic site are multiplied by crystal symmetry according to their Wyckoff position (see below) and are all related to each other. It is basically the same tensor with different orientations within the crystal lattice, which for the chemical shift tensor means that the isotropic chemical shift $\delta_{\mathrm{iso}}$ (the scaled trace of the tensor, which is invariant under transformations) is identical for all symmetry-related sites. For a given orientation of the magnetic field vector $\vec{b}$ in the crystal lattice, symmetry-related tensors may still give rise to distinguishable signals for a single crystal because their relative orientation to $\vec{b}$ may differ. In a powder sample, orientations become degenerate, and this distinguishability of magnetic non-equivalent sites is lost. In contrast, for tensors related by either inversion or translation elements, which renders them *magnetically equivalent*, their resonances always show up in identical positions, even in a single crystal. For interaction tensors belonging to the same nuclide but situated on distinct crystallographic sites, no such relations need to exist, and for the chemical shift, different values of $\delta_{\mathrm{iso}}$ are possible.

As a specific example, let us consider the Pb atoms in the crystal structure of the natural mineral vanadinite, Pb_5_(VO_4_)_3_Cl, as listed in Table 4. From the space group charts in the International Tables for Crystallography [109], the coordinates of the crystallographically equivalent sites generated by the symmetry elements for a given Wyckoff position can be obtained. There are six such sites for position 6h, and four for 4f:

(35) $$\begin{array}{cccc} 6h\; & \left(1\right)\;x,y,\frac{1}{4}\; & \left(2\right)\;\bar{y},x-y,\frac{1}{4}\; & \left(3\right)\;\bar{x}+y,\bar{x},\frac{1}{4}\\ \; & \left(4\right)\;\bar{x},\bar{y},\frac{3}{4}\; & \left(5\right)\;y,\bar{x}+y,\frac{3}{4}\; & \left(6\right)\;x-y,x,\frac{3}{4}, \end{array}\;\begin{array}{ccc} 4f\; & \left(1\right)\;\frac{1}{3},\frac{2}{3},z\; & \left(2\right)\;\frac{2}{3},\frac{1}{3},z+\frac{1}{2}\\ \; & \left(3\right)\;\frac{2}{3},\frac{1}{3},\bar{z}\; & \left(4\right)\;\frac{1}{3},\frac{2}{3},\bar{z}+\frac{1}{2}. \end{array}$$

It can be seen that for position 6h, the coordinates of (1,4), (2,5), and (3,6) are pairwise-connected by an inversion; the same is true for pairs (1,3) and (2,4) for Wyckoff position 4f. According to the above definition, these pairs are magnetically equivalent and will, hence, always give rise to identical resonance frequencies. Alternatively, this can be understood to be a consequence of the fact that second-rank tensors are always invariant under inversion. This can be demonstrated by considering a general (symmetric) chemical shift tensor in an orthogonal crystal coordinate system abc, associated with the Wyckoff site (1)x,y,z:

(36) $$\delta_{\left(1\right)}=\left(\begin{array}{ccc} \delta_{aa} & \delta_{ab} & \delta_{ac}\\ \delta_{ab} & \delta_{bb} & \delta_{bc}\\ \delta_{ac} & \delta_{bc} & \delta_{cc} \end{array}\right).$$

Inversion symmetry creates the site $\left(2\right)\;\bar{x},\bar{y},\bar{z}$ from site (1)x,y,z. The matrix performing this transformation is just the negative identity matrix $R_{\mathrm{inv}}=-E$, and the chemical shift tensor $\delta_{\left(2\right)}$ at site (2) is generated from that at site (1) by the transformation (see also Equation (A8)):

(37) $$\begin{array}{ccc} \delta_{\left(2\right)} & = & R_{\mathrm{inv}}^{-1}\delta_{\left(1\right)}R_{\mathrm{inv}}\\ \; & = & \left(\begin{array}{ccc} -1 & 0 & 0\\ 0 & -1 & 0\\ 0 & 0 & -1 \end{array}\right)\left(\begin{array}{ccc} \delta_{aa} & \delta_{ab} & \delta_{ac}\\ \delta_{ab} & \delta_{bb} & \delta_{bc}\\ \delta_{ac} & \delta_{bc} & \delta_{cc} \end{array}\right)\left(\begin{array}{ccc} -1 & 0 & 0\\ 0 & -1 & 0\\ 0 & 0 & -1 \end{array}\right)\\ \; & = & \left(\begin{array}{ccc} \delta_{aa} & \delta_{ab} & \delta_{ac}\\ \delta_{ab} & \delta_{bb} & \delta_{bc}\\ \delta_{ac} & \delta_{bc} & \delta_{cc} \end{array}\right). \end{array}$$

Since $\delta_{\left(2\right)}=\delta_{\left(1\right)}$, the two tensors will always produce identical resonance frequencies, no matter what their relative orientation to the external magnetic field is. For symmetry elements other than inversion or translation, the situation is different. At the 4f site of ^207^Pb in vanadinite, positions (1,4) and (2,3) are connected by a mirror plane with its plane normal along *c*. With the relevant transformation matrix $S_{c}$ given by Equation (A10), the chemical shift tensor at site (4) is generated from site (1) by the following:

(38) $$\begin{array}{ccc} \delta_{\left(4\right)} & = & S_{c}^{-1}\delta_{\left(1\right)}S_{c}\\ \; & = & \left(\begin{array}{ccc} 1 & 0 & 0\\ 0 & 1 & 0\\ 0 & 0 & -1 \end{array}\right)\left(\begin{array}{ccc} \delta_{aa} & \delta_{ab} & \delta_{ac}\\ \delta_{ab} & \delta_{bb} & \delta_{bc}\\ \delta_{ac} & \delta_{bc} & \delta_{cc} \end{array}\right)\left(\begin{array}{ccc} 1 & 0 & 0\\ 0 & 1 & 0\\ 0 & 0 & -1 \end{array}\right)\\ \; & = & \left(\begin{array}{ccc} \delta_{aa} & \delta_{ab} & -\delta_{ac}\\ \delta_{ab} & \delta_{bb} & -\delta_{bc}\\ -\delta_{ac} & -\delta_{bc} & \delta_{cc} \end{array}\right). \end{array}$$

The mirror plane induces a sign change in some of the off-diagonal elements, $\delta_{\left(4\right)}\neq \delta_{\left(1\right)}$; therefore, for some orientations of the magnetic field vector, the resonance position determined by $\delta_{\left(4\right)}$ may be different from that produced by $\delta_{\left(1\right)}$, hence, the two sites are not magnetically equivalent. It should be noted that the components of the two tensors (4) and (1) still have the same magnitude, and only differ by sign (i.e., tensor orientation). In the NMR spectra of polycrystalline samples (Figure 8), magnetically inequivalent sites are not distinguishable due to the orientation degeneracy, and under MAS, produce identical isotropic resonances, i.e., for the 4f site of ^207^Pb in vanadinite, $\delta_{\left(4\right)}^{\mathrm{iso}}=\delta_{\left(1\right)}^{\mathrm{iso}}$. A similar situation exists for the 6h position in vanadinite. Here, sites (1,2,3) and (4,5,6) are connected by a six-fold screw-axis, which also leads to sign changes for individual tensors. Because of pairing by inversion, three distinguishable ^207^Pb resonances may be observed from a single crystal, but the spectrum of a polycrystalline sample shows again only a single site, see Figure 8. Since NMR is not sensitive to translations, screw-axes can be treated as pure rotations (see below for an example); similarly, the action of glide planes can be reduced to mirror planes. A related discussion of the action of symmetry elements on NMR tensors may be found in review papers by Kennedy and Ellis [111,112].

### 4.3. Effects of Crystal Symmetry on Tensor Shape

The actual shape of the interaction tensors in a periodic solid is strongly dependent on the symmetry elements present in the crystal structure. The basic principle is that the tensor must conform to the symmetry of the site it is placed on. If, for example, the symmetry element happens to be a rotation (or screw) axis, the tensor is not allowed to change its shape under rotational transformation, which, for *n*-fold axes with n≥3, imposes uniaxiality ($\delta_{11}=\delta_{22}$) on it.

An extreme case is provided by wulfenite, PbMoO_4_. In its crystal structure [113], only one magnetically inequivalent ^207^Pb site exists, placed on Wyckoff position 4a in the tetragonal unit cell. The four ^207^Pb atoms belonging to 4a can be understood as being generated by a series of inversion operations, which renders all of them magnetically equivalent. Some of these inversion operations are due to four-fold roto-inversion axes ($\bar{4}$) parallel to the crystallographic *c*-axis, on which the tensors are actually placed. Applying a single $90^{\circ }$ rotation around *c* to our test tensor $\delta_{\left(1\right)}$ in the abc system provides the following:

(39) $$\begin{array}{ccc} \delta (90^{\circ }) & = & R_{c}^{-1}\left(90^{\circ }\right)\delta_{\left(1\right)}R_{c}\left(90^{\circ }\right)\\ \; & = & \left(\begin{array}{ccc} 0 & +1 & 0\\ -1 & 0 & 0\\ 0 & 0 & +1 \end{array}\right)\left(\begin{array}{ccc} \delta_{aa} & \delta_{ab} & \delta_{ac}\\ \delta_{ab} & \delta_{bb} & \delta_{bc}\\ \delta_{ac} & \delta_{bc} & \delta_{cc} \end{array}\right)\left(\begin{array}{ccc} 0 & -1 & 0\\ +1 & 0 & 0\\ 0 & 0 & +1 \end{array}\right)\\ \; & = & \left(\begin{array}{ccc} \delta_{bb} & -\delta_{ab} & \delta_{bc}\\ -\delta_{ab} & \delta_{aa} & -\delta_{ac}\\ \delta_{bc} & -\delta_{ac} & \delta_{cc} \end{array}\right). \end{array}$$

Because the form of the chemical shift tensor needs to be invariant against the above transformation, it follows that $\delta_{\left(1\right)}$ must (a) be uniaxial, $\delta_{aa}=\delta_{bb}$, and since finite numerical values on the off-diagonal elements $\delta_{ij}$ would have to change their signs and/or values, (b) have all $\delta_{ij}$ equal to zero. Therefore, the CRY frame constitutes the principal axes system of this tensor, $\delta^{\mathrm{CRY}}=\delta^{\mathrm{PAS}}$. The chemical shift tensor of ^207^Pb in wulfenite is thus described by only two independent tensor components, which can be named $\delta_{aa}$ and $\delta_{cc}$, producing the simplest possible non-isotropic tensor:

(40) $$\begin{array}{cc} \; & \delta_{\mathrm{wulf}}^{\mathrm{CRY}}=\delta_{\mathrm{wulf}}^{\mathrm{PAS}}=\left(\begin{array}{ccc} \delta_{aa} & 0 & 0\\ 0 & \delta_{aa} & 0\\ 0 & 0 & \delta_{cc}. \end{array}\right)\; \end{array}$$

In general, interaction tensors T subjected to symmetry restraints have less independent components $t_{\mathrm{idp}}$ than those without restraints. For the (symmetric) chemical shift tensor, unrestrained means $t_{\mathrm{idp}}=6$, and for the dipolar and quadrupole coupling tensors, $t_{\mathrm{idp}}=5$, because they are traceless, $t_{11}+t_{22}+t_{33}=0$. A reduced set of tensor components usually simplifies tensor determination by single-crystal NMR, as discussed below.

## 5. Recording the Orientation Dependence: The Rotation Pattern

### 5.1. Goniometer Axis Perpendicular to the Magnetic Field

**Figure 9:**
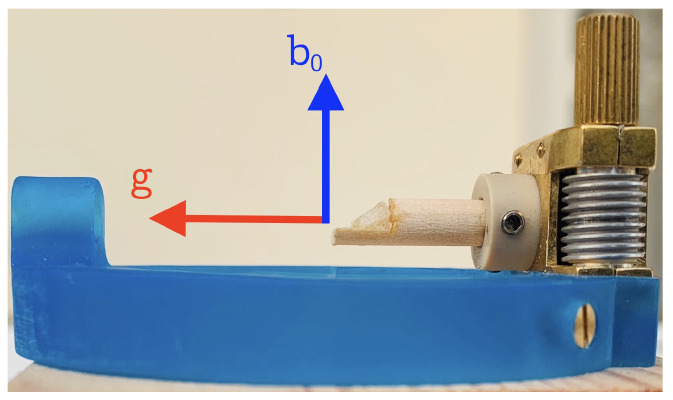
Goniometer mechanics with the rotation axis *g* (red) perpendicular to the external magnetic field $b_{0}$ (blue). Defined orientation change is effected by a worm gear, with the worm wheel not being visible. The rotation axis on which the single crystal is mounted has a diameter of 5 mm. For actual NMR measurements, an RF coil needs to be added to this setup (mechanics built by NMR Service GmbH, Erfurt, Germany).

**Figure 10:**
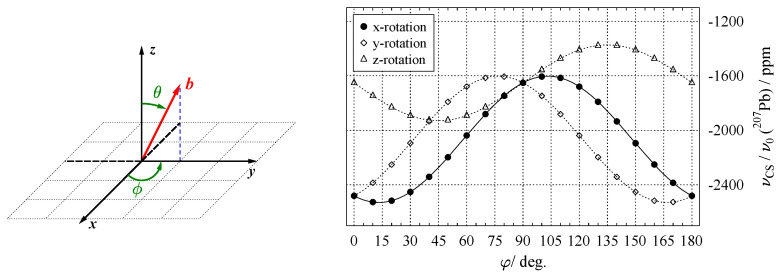
(**Left**): Spherical coordinates for a rotation of the magnetic field vector *b* around the *x*-axis. (**Right**): Resulting rotation patterns for the three rotations around *x*, *y*, and *z*, with the rotation (goniometer) axis always perpendicular to the external magnetic field, using the ^207^Pb chemical shift data of phosgenite (Equation (59)) as an example.

To record the orientation dependence of NMR interactions, a single crystal is mounted on goniometer mechanics, such as the one shown in Figure 9. In most cases, the rotation axis of the goniometer is perpendicular to the external magnetic field (see below for alternative setups). Several NMR spectra are now acquired for a series of defined crystal orientations, with the plot of resonance positions over the rotation angle being called a *rotation pattern*. To illustrate this procedure in more detail, we will look at a single atomic site in a unit cell, occupied by a nuclide with spin I=1/2, where the only relevant orientation dependence is caused by the chemical shift. In an arbitrary coordinate system xyz, the chemical shift tensor δ has the form shown in Equation (32), and the magnetic field vector $\vec{b}$ (Equation (31)) adopts a general orientation. We now assume that we are capable of orienting our single crystal under investigation such that initially, the xyz frame is fully aligned with the LAB frame (where $\vec{b}$ points along the *z*-axis). If the *x*-axis of the xyz-frame is chosen as a rotation (goniometer) axis, the field vector will move in the *y*–*z* plane by the specified rotation angle $\varphi_{i}$. From the left-hand side of Figure 10, it is evident that for this rotation, the azimuthal angle is always $\phi =90^{\circ }$, and $\theta \to \varphi_{i}$.

Therefore, the field vector now depends on $\varphi_{i}$ in the following:

(41) $${\vec{b}}_{yz}=\left(\begin{array}{c} 0\\ \sin\varphi_{i}\\ \cos\varphi_{i} \end{array}\right).$$

Following Equation (32), the resonance position is calculated as follows:

(42) $$\nu_{CS}\left(\varphi_{i}\right)=\left(\begin{array}{ccc} 0 & \sin\varphi_{i} & \cos\varphi_{i} \end{array}\right)\cdot \left(\begin{array}{ccc} \delta_{xx} & \delta_{xy} & \delta_{xz}\\ \delta_{xy} & \delta_{yy} & \delta_{yz}\\ \delta_{xz} & \delta_{yz} & \delta_{zz} \end{array}\right),\cdot \left(\begin{array}{c} 0\\ \sin\varphi_{i}\\ \cos\varphi_{i} \end{array}\right).$$

Carrying out these multiplications leads to the following:

(43) $$\begin{array}{ccc} \nu_{CS}\left(\varphi_{i}\right) & = & \delta_{yy}\sin^{2}\varphi_{i}+\delta_{yz}\sin\varphi_{i}\cos\varphi_{i}+\\ \; & \; & \delta_{yz}\sin\varphi_{i}\cos\varphi_{i}+\delta_{zz}\cos^{2}\varphi_{i} \end{array}.$$

To recast the above expression, the following trigonometric relations are needed:

(44) $$\begin{array}{ccc} \sin^{2}\varphi & = & \frac{1}{2}\left(1-\cos2\varphi \right)\\ \cos^{2}\varphi & = & \frac{1}{2}\left(1+\cos2\varphi \right)\\ \sin2\varphi & = & 2\sin\varphi \cos\varphi \end{array}.$$

The final form of the dependence of the resonance position on the rotation angle $\varphi_{i}$ around the *x*-axis of the xyz-frame is then as follows:

(45) $$\nu_{CS}\left(\varphi_{i}\right)=\frac{\left(\delta_{zz}+\delta_{yy}\right)}{2}+\frac{\left(\delta_{zz}-\delta_{yy}\right)}{2}\cos2\varphi_{i}+\delta_{yz}\sin2\varphi_{i}.$$

Using the experimentally determined values for the chemical shift of ^207^Pb in phosgenite (see below, Equation (59)), the data points measured in steps of $\Delta \varphi_{i}=10^{\circ }$ for this hypothetical ‘perfect’ rotation around *x*, together with the resulting harmonic function (Equation (45)), are plotted on the right-hand-side of Figure 10. Since NMR is not sensitive to inversion (see Equation (37)), it is sufficient to record such rotation patterns for the range $\varphi_{i}=0\ldots 180^{\circ }$, and not full circle. For the situation discussed here, i.e., rotating a second-rank tensor around an axis, which is perpendicular to the magnetic field, the resulting NMR response is always a harmonic function of the type derived above. The general form of these harmonics was first reported by Volkoff et al. [4]:

(46) $$\nu_{⊥}\left(\varphi_{i}\right)=A+B\cos2\varphi_{i}+C\sin2\varphi_{i}.$$

The right-hand-side of Figure 10 also shows the data points and harmonic functions (dashed lines) resulting from rotating the crystal around the *y*- and *z*-axis. These additional data are needed to determine the full tensor, as the standard method for extracting a tensor from a single crystal is to acquire data for three rotation axes which are all orthogonal to each other, as will be explained in more detail below.

### 5.2. Other Goniometer Geometries

In case the goniometer axis is oriented not exactly perpendicular to the external magnetic field, the observed harmonics contain not just terms depending on the double angle $2\varphi_{i}$, as in Equation (46), but also on $\varphi_{i}$:

(47) ν⊥φi=A+Bcos2φi+Csin2φi+Dcosφi+Esinφi.

Vosegaard et al. [114] built a goniometer with the rotation axis tilted by $45^{\circ }$ to the external magnetic field. For such a goniometer orientation, it is possible to extract a complete tensor using only two different, non-orthogonal rotation axes, which are realised by mounting the single crystal in two different orientations on the $45^{\circ }$-axis. Even fewer orientation-dependent data are needed for the ‘multiple-axis flipper probe’ constructed by Grant and co-workers [115]. Here, only six carefully chosen orientations of a crystal are measured and analysed to obtain the complete tensor. However, this technique involves acquisition of 2D chemical-shift correlation spectra, designed to detect correlations between these six special orientations. Thus, some of the time saved by having to measure less orientations has to be re-invested in spectrometer time for recording 2D spectra. A comprehensive discussion of goniometer geometries and other hardware-related issues may be found in a recent review [12].

## 6. Analysing the Orientation Dependence: From Rotation Pattern to Tensor

The first task of data analysis after acquisition of a rotation pattern is to sort the observed resonances into groups which belong to a single harmonics $\nu_{j}\left(\varphi_{i}\right)$ of the type shown in Equation (46). This sorting procedure can be straightforward, as for the situation depicted in Figure 10. However, if several harmonics are present in a rotation pattern, and overlapping resonances or crossings of these harmonics are encountered, it may become problematic. Conflicts can usually be resolved by changing the assignment of the questionable data point and comparing the resulting goodness-of-fit parameters, with the correct assignment giving the better parameter. An automated sorting algorithm has been suggested by Heuer [116]. The concept behind this is to search in the abstract space spanned by coefficients *A*, *B*, and *C* of Equation (46), which uniquely define each harmonic function $\nu_{j}\left(\varphi_{i}\right)$ connecting the data points. In ABC-space, every harmonic function in the rotation pattern is represented by a single point $h_{j}$. By dividing the ABC-space into blocks, searching these blocks for existing $h_{j}$, and then successively reducing the block size, valid solutions for the $\nu_{j}\left(\varphi_{i}\right)$ may be found.

Once the data points have been assigned to their respective harmonics (and the harmonics, if necessary, to the symmetry related sites of the observed nuclide), the data can be evaluated to extract the tensor values. Obviously, this process can greatly benefit from the application of computer routines. Over the years, several such algorithms have been implemented and published, the most prominent being the program Asics (Analysis of SIngle-Crystal Spectra) by Vosegaard [117], which has also been made available as the internet-based tool *web*Asics [118]. Another recent addition is the software SCFit by Xu and Bryce [119], which allows restrained data fitting using tensor values from different sources, such as powder spectra. The program Superfit was created by Tegenfeldt [120], and used extensively in the group of Ulrich Haeberlen in Heidelberg [77]. Also, distributed among all the research groups involved in single-crystal NMR, there must exist many lines of clever computer code written and tested over the years, which have never been made publicly available. With the strategies outlined below, it is possible to perform satisfactory data analysis using commercially available software. However, since tensor components may have an effect on several harmonics in one rotation pattern (see below), the software should allow fitting several (x,y) functions to the same fit parameter set. This is achievable with advanced analysis software such as Igor Pro (WaveMetrics, Portland, OR, USA).

### 6.1. Chemical Shift Tensor

Extracting the components of the chemical shift tensor is just the reverse of the procedure explained in Section 5.1. The data points of the recorded rotation pattern are fitted to a Volkoff harmonics (Equation (46)) in order to extract the coefficients *A*, *B*, and *C*. For the rotation around the *x*-axis (solid line in Figure 10), these prefactors are related to the tensor components in the following way:

(48) $$\begin{array}{ccc} A_{x} & = & \frac{\left(\delta_{zz}+\delta_{yy}\right)}{2},\\ B_{x} & = & \frac{\left(\delta_{zz}-\delta_{yy}\right)}{2},\\ C_{x} & = & \delta_{yz}. \end{array}$$

From these three equations we obtain the values of the three unknowns: $\delta_{yy}$, $\delta_{yz}$, and $\delta_{zz}$. A general (symmetric) chemical shift tensor, however, possesses $t_{idp}=6$ independent components. To determine the remaining components, additional rotation patterns around different goniometer axes need to be recorded. To avoid overlap of data, the additional axes should be perpendicular to the first one. The rotation patterns resulting from having the goniometer along the *y*- and *z*-axis are also shown in Figure 10. Analysing them in the same way as the data for the *x*-axis, i.e., applying a fit to the data points to extract the coefficients, leads to the following result:

(49) $$\begin{array}{ccccccc} A_{y} & = & \frac{\left(\delta_{xx}+\delta_{zz}\right)}{2}, & \;\; & A_{z} & = & \frac{\left(\delta_{xx}+\delta_{yy}\right)}{2},\\ B_{y} & = & \frac{\left(\delta_{zz}-\delta_{xx}\right)}{2}, & \;\; & B_{z} & = & \frac{\left(\delta_{xx}-\delta_{yy}\right)}{2},\\ C_{y} & = & \delta_{xz}, & \;\; & C_{z} & = & \delta_{xy}. \end{array}$$

After solving the above equations for the still missing elements $\delta_{ij}$, the chemical shift tensor under investigation is now completely determined. This is the reasoning behind the concept of recording three independent rotation patterns when studying single crystals by NMR [4], which has also influenced the development of hardware. For example, special sample holders have been constructed, which ensure the orthogonality of the three used axes [117]. It also should be emphasised that for the above procedure to work, the orientation of the single crystal with respect to the three goniometer axes needs to be known precisely, including the initial direction of the crystal. All deviations from the ideal situation lead to higher complexity of the fitting routines, as additional parameters may have to be introduced, such as an offset angle for the uncertainty of the starting position.

However, by inspecting Equations (48) and (49), it may also be seen that our model system is overdetermined; for example, the value of $\delta_{xx}$ is encapsulated in the coefficients $A_{y}$, $B_{y}$, $A_{z}$, and $B_{z}$. To put this argument on a more quantitative footing, the number of independent tensor components that should be determined ($t_{\mathrm{idp}}$) needs to be compared to the number of parameters available from the experiment ($p_{\exp}$). Obviously, from fitting a single harmonics to Equation (46), such as the solid-line fit shown in Figure 10, we obtain three parameters (*A*, *B*, and *C*); that is, $p_{\exp}=3$. However, the seemingly logical extension of getting $p_{\exp}=6$ from fitting two harmonics is not valid [120,121,122,123]. This can be understood by the following argument [123]: consider a tensor T(1) in the crystal frame (CRY), and a second tensor T(2), which is generated from the first by the transformation $T\left(2\right)=K^{-1}T\left(1\right)K$, where the transformation matrix K could, for example, describe a general rotation, see Equation (A6). The tensor T(1) may be related to T(2) by either a symmetry element of the crystal structure (see Section 6.3 below), or by a known, defined reorientation of the crystal on the goniometer axis between recording two rotation patterns. In the CRY frame, the direction of the external magnetic field vector $\vec{b}\left(\varphi_{i}\right)$ is variable and different for each $\varphi_{i}$. For an experimental set-up where the goniometer axis is oriented perpendicular to the magnetic field, $\vec{b}\left(\varphi_{i}\right)$ traces out great circles on a unit sphere when recording a rotation pattern. Since any two great circles on a sphere intersect, there must be one angle $\varphi_{s}$ within a $180^{\circ }$ rotation interval where the two related tensors T(1) and T(2) produce identical line positions, respectively, splittings:

(50) $$\left(\Delta \right)\nu \left(\varphi_{s}\right)∼{\vec{b}}^{T}\left(\varphi_{s}\right)\cdot T\left(1\right)\cdot \vec{b}\left(\varphi_{s}\right)={\vec{b}}^{T}\left(\varphi_{s}\right)\cdot K^{-1}T\left(1\right)K\cdot \vec{b}\left(\varphi_{s}\right).$$

The two harmonics belonging to T(1) and T(2) are hence not completely independent but linked by one constraint, thus delivering $p_{\exp}=6-1=5$ parameters. The general balance of unknown tensor components $t_{\mathrm{idp}}$ versus experimentally available parameters $p_{\exp}$ for *r* distinct rotation patterns with different (known) goniometer axes may be written as follows:

(51) $$p_{\exp}=3r-\left(r-1\right)=2r+1\geq t_{\mathrm{idp}}.$$

For the chemical shift tensor discussed above, $t_{\mathrm{idp}}=6$. Therefore, recording only r=2 rotation patterns is not sufficient to determine the full tensor, but recording r=3 patterns leads to $p_{\exp}=7$, i.e., a slight overdetermination. This hints at the fact that the time-consuming recording and analysing of three full rotation patterns is perhaps not always necessary, as will be discussed further below.

### 6.2. Quadrupole Coupling Tensor

**Figure 11:**
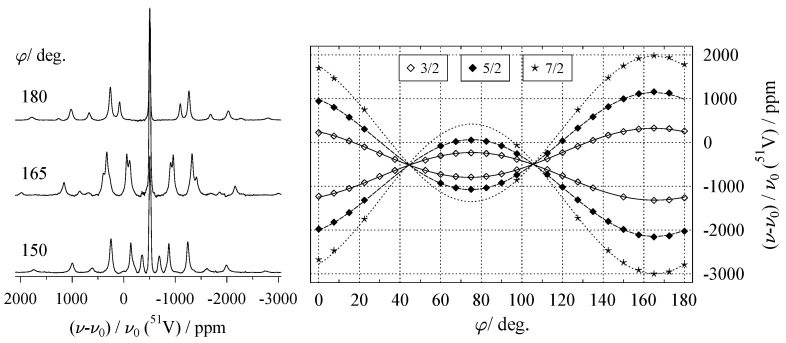
Single-crystal NMR of vanadinite, Pb_5_(VO_4_)_3_Cl. (**Left**): Spectra of ^51^V (I=7/2) at the indicated nominal rotation angles φ. The central transitions are plotted off scale to improve visibility of the satellite transitions. (**Right**): Rotation pattern over the range of $\varphi =0\ldots 180^{\circ }$ for one of the three magnetically equivalent ^51^V pairs in the crystal structure, designated V(3) in the original publication [124].

When dealing with the NMR spectra of nuclides with spin I>1/2, the simultaneous presence of more than one NMR interaction needs to be considered. When the contributions of the dipolar interaction can be neglected (as it may in most cases), the resonance positions are affected by (a) the quadrupolar interaction, and (b) the chemical shift. Usually, the quadrupolar interaction is much stronger than the chemical shift and, therefore, generates a more pronounced orientation dependence. Each of the 2I transitions with its associated parameter *k* now possesses its own harmonic function, as depicted in Figure 11. When second-order quadrupole contributions (see Equation (10)) are present, additional terms to those of Equation (46) have to be included in the harmonic functions:

(52) $$\nu_{⊥}^{k}\left(\varphi_{i}\right)=A^{k}+B^{k}\cos2\varphi_{i}+C^{k}\sin2\varphi_{i}+G^{k}\cos4\varphi_{i}+H^{k}\sin4\varphi_{i}.$$

Figure 11 shows the orientation dependence for the k=±1,±2,±3 satellite transitions for ^51^V with I=7/2 in the natural mineral vanadinite [124], where second-order effects (described by $G^{k}$ and $H^{k}$ in the above equation) are practically absent. (Fitting data points to Equation (52) and inspecting the magnitude of the resulting $G^{k}$ and $H^{k}$ coefficients provides a convenient test for the presence of second-order effects.) In general, to isolate the effect of quadrupole interaction to first-order, the differing sign dependence of the various interactions (see Table 2) can be made use of. Thus, by taking the difference between the $\nu_{+k}$ and $\nu_{-k}$ resonances of the satellite transitions shown in Figure 11, both the effects of chemical shift and second-order quadrupole are removed. Using the tensor notation of Equation (33), the evolution of $\Delta \nu (\varphi_{i})$ may be described as follows:

(53) $$\Delta \nu_{\pm k}\left(\varphi_{i}\right)=\nu_{+k}^{\left(1\right)}-\nu_{-k}^{\left(1\right)}=\frac{3\Delta k}{2I(2I-1)}{\vec{b}}^{T}\left(\varphi_{i}\right)\cdot Q\cdot \vec{b}\left(\varphi_{i}\right).$$

**Figure 12:**
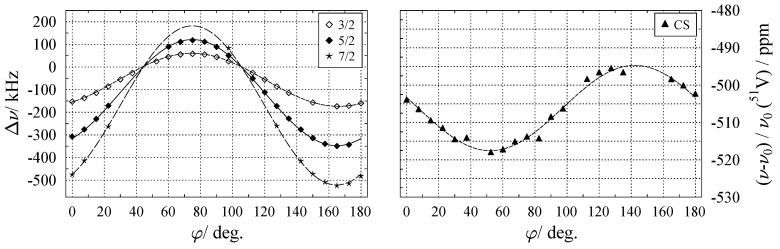
^51^V-NMR of vanadinite, Pb_5_(VO_4_)_3_Cl. (**Left**): Splittings Δν (Equation (53)) for the various satellite transitions, derived from the line positions displayed in Figure 11. (**Right**): Chemical shift evolution derived from plotting the centres $\nu^{\Delta k/2}$ (Equation (54)) of the 3/2 satellite transitions.

These splittings $\Delta \nu (\varphi_{i})$ are plotted on the left of Figure 12. Since the $\Delta \nu_{\pm k}$ for the various k=±1,±2,±3 satellite transitions are connected by a fixed factor, including all *k* into the data fit does not give inherently new information, but increases the number of data points, which, in turn, may improve the fit quality. In the case of ^51^V in vanadinite, the quadrupole interaction parameters come out to χ=2.52 MHz and $\eta_{Q}=0.52$, see Table A2.

Returning to the balance of independent tensor components to experimental parameters (Equation (51)), recording r=2 rotation patterns should be sufficient to obtain the traceless quadrupole coupling tensor with $t_{\mathrm{idp}}=5$. This is, in principle, correct; however, the two goniometer axes used for these two rotation patterns should not be orthogonal to each other, as this induces another, in this context unwanted, constraint [121,122,123].

With the quadrupolar interaction calculated, there remains the effect of the chemical shift on the resonance positions, which as for spin I=1/2, is described by a tensor. For a situation similar to that of ^51^V in vanadinite, i.e., where the quadrupolar coupling is not sufficiently strong enough to induce second-order effects, the influence of the chemical shift may be extracted by simply following the resonance position of the central transition (CT), see, for example, Reference [125]. For spin systems with appreciable quadrupole coupling, however, the CT moves under both chemical shift and quadrupolar second-order shift. To isolate the chemical shift effect, the second-order influence according to Equation (25) must be removed by subtracting it. This process requires the knowledge of the angles α and β in the respective coordinate system and may involve some cumbersome transformations. Even for systems where the quadrupole interaction is present only to first-order, such as ^51^V in vanadinite, the CT contributions of the non-equivalent sites may be not or only partially resolved in the spectra, as shown for vanadinite on the left of Figure 11. For such systems, the variation of the centres of the satellite transitions may be evaluated instead [126,127]. That is, in the absence of second-order effects, the following quantity is only affected by the chemical shift:

(54) $$\nu^{\Delta k/2}\left(\varphi_{i}\right)=\frac{\nu^{+k}+\nu^{-k}}{2}.$$

A plot of the $\nu^{\Delta k/2}$ for ^51^V in vanadinite can be seen on the right of Figure 12. After obtaining these harmonics for the satellite centres, the procedure of determining the chemical shift tensor is exactly the same as the one outlined above in Section 6.1.

### 6.3. The Single-Rotation Method

The first report on the ‘single-rotation method’ seems to have been published by John A. Weil in 1973 [108]. Afterwards, the concept was (re-)discovered several times by various authors [120,121,122,123]. The idea behind this method is to exploit the presence of crystallographically related tensors within the crystal structure, whose relative orientations are defined by the symmetry operations connecting them. At the same time, these tensors are magnetically non-equivalent (i.e., not connected by either inversion or translation), and they give rise to distinct resonances in the spectrum. This way, an interaction tensor multiplied for $m_{n}$ magnetically non-equivalent sites by crystal symmetry delivers $m_{n}$ distinct rotation patterns for a single physical rotation around a goniometer axis. An alternative view is to think about the $m_{n}$ observed harmonics as being recorded from virtual goniometer axes, which are generated from the original (physical) one by application of the same symmetry elements which multiply the tensors for the given Wyckoff position. Obviously, a given system may contain more than one Wyckoff position for the observed nuclide, where each position may have a distinct interaction tensor. For *n* Wyckoff positions with a respective magnetic multiplicity of $m_{n}$, overall $\sum_{n}m_{n}$ signals may be show up in the spectrum. Considering again the balance of unknown tensor components $t_{\mathrm{idp}}$ versus experimentally available parameters $p_{\exp}$ (see Equation (51)) for this situation, the full tensor may be determined from a single rotation pattern if the following condition is fulfilled:

(55) $$p_{\exp}^{r=1}=\sum_{n}\left(2m_{n}+1\right)\geq n\cdot t_{\mathrm{idp}}.$$

With $t_{\mathrm{idp}}=5$ for quadrupole coupling and $t_{\mathrm{idp}}=6$ for the chemical shift, it is immediately clear from the above relation that for Wyckoff positions possessing at least three-fold symmetry, the single-rotation method always delivers enough parameters to obtain the full tensor if the orientation of the goniometer axis $\vec{g}$ is known. The one caveat is that $\vec{g}$ should not be aligned with the symmetry element generating the $m_{n}$ multiplicity, i.e., oriented parallel to a rotation/screw axis or lying in a mirror plane. In this case, the resonance positions of the tensors connected by this symmetry element will become identical, and information is lost. A more fundamental discussion of this topic in terms of crystal rotation groups may be found in the early paper by Weil [108].

As a specific example of applying the single-rotation method, let us consider the ^207^Pb-NMR of the natural mineral phosgenite, Pb_2_Cl_2_CO_3_, which crystallises in the tetragonal space group P4/mbm [128]. In the unit cell, the Pb atoms reside at Wyckoff position 8*k* and form two sets of four atoms, which are related by a four-fold rotation axis parallel to the *c*-axis:

(56) $$\underset{\left(x,x+\frac{1}{2},z\right)}{\mathrm{Pb}(1)}\overset{90^{\circ }}{↔}\;\underset{\left(\bar{x}+\frac{1}{2},x,z\right)}{\mathrm{Pb}(2)}\overset{90^{\circ }}{↔}\;\underset{\left(\bar{x},\bar{x}+\frac{1}{2},z\right)}{\mathrm{Pb}(3)}\overset{90^{\circ }}{↔}\;\underset{\left(x+\frac{1}{2},\bar{x},z\right)}{\mathrm{Pb}(4)}.$$

**Figure 13:**
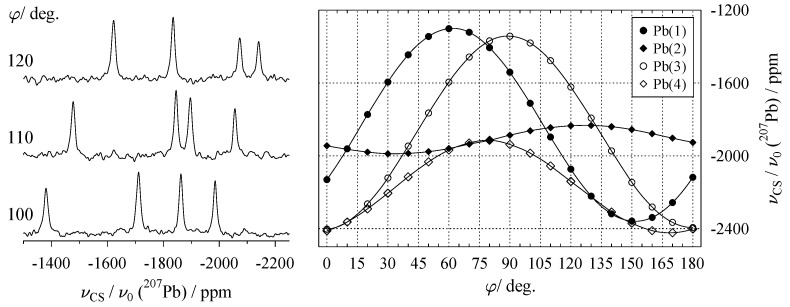
Single-crystal NMR of Phosgenite, Pb_2_Cl_2_CO_3_. **Left**: ^207^Pb-NMR spectra at the indicated nominal rotation angles φ. **Right**: Rotation pattern over the range of $\varphi =0-180^{\circ }$.

The two sets are connected to each other by an inversion center at the center of the unit cell, making them pairwise magnetically equivalent, so that only four distinct resonances are observed in the ^207^Pb-NMR spectra, as may be seen from Figure 13.

Because of the symmetry elements in the unit cell, the chemical shift tensors of the four distinct ^207^Pb in the tetragonal crystal frame (CRY) are described by only four independent tensor components, which may be designated as *P*, *Q*, *R*, and *S* [129]:

(57) $$\begin{array}{c} \begin{array}{cc} \; & \delta_{\mathrm{Pb}(1)}^{\mathrm{CRY}}=\left(\begin{array}{ccc} P & Q & R\\ Q & P & -R\\ R & -R & S \end{array}\right)\;\delta_{\mathrm{Pb}(2)}^{\mathrm{CRY}}=\left(\begin{array}{ccc} P & -Q & -R\\ -Q & P & -R\\ R & -R & S \end{array}\right)\\ \; & \delta_{\mathrm{Pb}(3)}^{\mathrm{CRY}}=\left(\begin{array}{ccc} P & Q & -R\\ Q & P & R\\ -R & R & S \end{array}\right)\;\delta_{\mathrm{Pb}(4)}^{\mathrm{CRY}}=\left(\begin{array}{ccc} P & -Q & R\\ -Q & P & R\\ R & R & S \end{array}\right) \end{array} \end{array}$$

With ^207^Pb in the phosgenite structure occupying the n=1 Wyckoff position with a magnetic multiplicity of $m_{n}=4$, the expression of Equation (55) reduces to the following:

$$p_{\exp}^{r=1}=2\cdot 4+1=9.$$

This is more than enough to extract a full chemical shift tensor with $t_{\mathrm{idp}}=6$ from the data shown in Figure 13, and even more so since for phosgenite $t_{\mathrm{idp}}=4$. The ^207^Pb chemical shift tensors displayed in Equation (57) are generated by crystal symmetry. While all four share the same components PQRS, the signs of the components change between tensors, reflecting their varying orientations in the crystal frame. Applying the principle of Equation (32), the data fit equation for tensor $\delta_{\mathrm{Pb}(1)}^{\mathrm{CRY}}$ can be written as follows:

(58) $$\begin{array}{ccc} \frac{\nu_{\mathrm{Pb}(1)}\left(\varphi_{i}\right)}{\nu_{0}} & = & {\vec{b}}_{0}^{T}\left(\varphi_{i}\right)\cdot \delta_{\mathrm{Pb}(1)}^{\mathrm{CRY}}\cdot {\vec{b}}_{0}\left(\varphi_{i}\right)\\ \; & = & P\left(b_{x}b_{x}+b_{y}b_{y}\right)+Q\left(2b_{x}b_{y}\right)+R\left(2b_{x}b_{z}-2b_{y}b_{z}\right)+S\left(b_{z}b_{z}\right). \end{array}$$

The other three tensors $\delta_{\mathrm{Pb}(2\ldots 4)}^{\mathrm{CRY}}$ produce expressions similar to Equation (58), but with a varying sign pattern. Before running the data fit, the harmonics shown in Figure 13 need to be assigned to different lead sites Pb(1…4) in the crystal structure. This can be done by symmetry considerations (e.g., what is the orientation of Pb(2) after generating it from Pb(1) by a $90^{\circ }$ rotation about the *c*-axis), or by a simple trial-and-error strategy. After correct assignment, a simultaneous fit of all four harmonics gives the following result for $\delta_{\mathrm{Pb}(1)}^{\mathrm{CRY}}$ (in ppm), with all other tensors easily derived by applying the signs of Equation (57):

(59) $$\begin{array}{ccc} \delta_{\mathrm{Pb}(1)}^{\mathrm{CRY}}=\left(\begin{array}{ccc} -1651.3 & -277.9 & 206.6\\ -277.9 & -1651.3 & -206.6\\ 206.6 & -206.6 & -2481 \end{array}\right)\; & \overset{\Omega }{⟷} & \delta_{\mathrm{Pb}(1)}^{\mathrm{PAS}}=\left(\begin{array}{ccc} -2553 & 0 & 0\\ 0 & -1929 & 0\\ 0 & 0 & -1301 \end{array}\right). \end{array}$$

In phosgenite (and similar systems), the excess of experimental parameters, $p_{\exp}^{r=1}>t_{\mathrm{idp}}$, makes it possible to include also the orientation of the goniometer axis itself into the data fit, as will be described in the following.

### 6.4. The Minimal-Rotation Method

The strategies outlined for the analysis of rotation patterns so far, i.e., the classical method of using three orthogonal goniometer axes (Section 6.1), or the single-rotation method (Section 6.3), require precise knowledge of the goniometer axis orientation. This pre-alignment of the single crystal on the goniometer may be achieved using X-ray diffraction or optical reflection methods. For crystals not amenable to these methods because they exhibit an irregular shape and/or high X-ray absorption, an alternative strategy may be of interest, namely fitting the goniometer axis direction directly from the NMR data [78,124,129,130]. In spherical coordinates, the goniometer vector $\vec{g}$ is expressed as follows:

(60) $$\vec{g}=\left(\begin{array}{c} \sin\theta_{g}\cos\phi_{g}\\ \sin\theta_{g}\sin\phi_{g}\\ \cos\theta_{g} \end{array}\right).$$

The angles $\theta_{g}$ and $\phi_{g}$ are now included as variables when fitting the rotation pattern, plus an offset angle $\varphi_{0}$ allowing for a variation of the starting point between theoretical description and experimental points. Therefore, the balance of Equation (55) must change to include three more independent variables per distinct rotation pattern *r*:

(61) $$p_{\exp}^{\min}=r\sum_{n}\left(2m_{n}+1\right)\geq \left(n\cdot t_{\mathrm{idp}}+3r\right).$$

In many cases, a single rotation pattern (r=1) might still be sufficient, but depending on the magnetic multiplicity $m_{n}$ of the *n* Wyckoff positions in the crystal lattice, data from additional goniometer axes might be needed, which is why this approach might be termed the ‘minimal-rotation method’.

**Figure 14:**
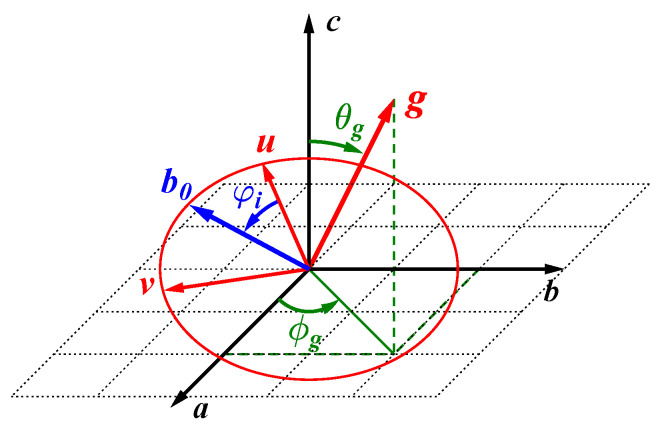
Description of the rotation of the magnetic field vector *b* around the goniometer axis *g* in the coordinate system abc using the two two auxiliary unit vectors *u* and *v*, see text for details.

When making the goniometer orientation a variable of the data fit, it makes sense to directly link the description of the magnetic field vector $\vec{b}$ to that of $\vec{g}$. Thus, to express the step-wise movement of $\vec{b}\left(\varphi_{i}\right)$ around $\vec{g}$, two auxiliary unit vectors, $\vec{u}$ and $\vec{v}$, may be defined in the plane perpendicular to $\vec{g}$, as depicted in Figure 14. Choosing the CRY frame as reference, and allowing the recorded data to offset from the ideal starting point by the angle $\varphi_{0}$, the orientation of $\vec{b}$ may be calculated from the following:

(62) $$\begin{array}{c} \vec{b}\left(\varphi_{i}\right)=\vec{v}\sin\left(\varphi_{i}-\varphi_{0}\right)+\vec{u}\cos\left(\varphi_{i}-\varphi_{0}\right). \end{array}$$

To define $\vec{u}$ and $\vec{v}$, an arbitrary reference vector is needed, which needs to be non-parallel to $\vec{g}$. With $\vec{c}=\left(\begin{array}{ccc} 0 & 0 & 1 \end{array}\right)$ chosen as reference, $\vec{u}$ and $\vec{v}$ are given by the following:

(63) $$\begin{array}{c} \begin{array}{cc} \vec{v}=\frac{1}{\sin\theta_{g}}\left(\vec{g}\times \vec{c}\right);\;\vec{u}=\vec{v}\times \vec{g} & =\frac{1}{\sin\theta_{g}}\left(\vec{g}\times \vec{c}\right)\times \vec{g}\\ \; & =\frac{1}{\sin\theta_{g}}\left(\vec{c}-\vec{g}\cos\theta_{g}\right). \end{array} \end{array}$$

For systems where the goniometer happens to be too close to the crystallographic *c*-axis, an alternative choice is needed. Using $\vec{b}=\left(\begin{array}{ccc} 0 & 1 & 0 \end{array}\right)$ instead of $\vec{c}$ as reference leads to the following:

(64) $$\begin{array}{c} \begin{array}{c} \vec{v}=\frac{1}{\sqrt{\cos^{2}\;\theta_{g}+\sin^{2}\;\theta_{g}\cos^{2}\;\phi_{g}}}\left(\vec{g}\times \vec{b}\right);\;\\ \vec{u}=\vec{v}\times \vec{g}=\frac{1}{\sqrt{\cos^{2}\;\theta_{g}+\sin^{2}\;\theta_{g}\cos^{2}\;\phi_{g}}}\left(\vec{g}\times \vec{b}\right)\times \vec{g}\\ =\frac{1}{\sqrt{\cos^{2}\;\theta_{g}+\sin^{2}\;\theta_{g}\cos^{2}\;\phi_{g}}}\left(\vec{b}-\vec{g}\sin\theta_{g}\sin\phi_{g}\right). \end{array} \end{array}$$

Independent of the choice of $\vec{u}$ and $\vec{v}$, the movement of the magnetic field vector is described by Equation (62) for every rotation angle $\varphi_{i}$. With this expression substituted into the fit equations of the type shown in Equation (58), the components of symmetry related tensors may now be fitted simultaneously, with the fit algorithm determining the goniometer orientation (as described by the variables $\theta_{g}$, $\phi_{g}$ and $\varphi_{0}$) at the same time. For many systems, such as the phosgenite data shown in Figure 13, such fits converge nicely. (Since phosgenite crystals deliver well-defined and actually overdetermined data for ^207^Pb-NMR, it has been suggested to use them as an internal reference for goniometer axis determination when measuring less well suited systems [129].) However, for systems with comparatively high symmetry, ambiguities may remain in the sense that several solutions of equal quality (as defined by the fit residues) exist. In some cases, these ambiguities may be resolved by additional considerations, such as examining intersection points of harmonics (see below) or evaluating the orientation of external crystal surfaces. Where this is not possible, one may have to resort again to pre-alignment of the crystal.

A similar approach to derive the goniometer orientation from NMR data only, closely related to that outlined above, has been suggested by Harbison and co-workers [131]. Here, the information provided by intersection points of the harmonic functions (see Equation (50)) in the rotation pattern is exploited to derive information about the direction of the goniometer axis. Such intersection points occur when the magnetic field vector happens to point along a symmetry axis or lies in a symmetry plane, rendering the tensors connected by these symmetry elements magnetically equivalent. For these intersection points, a set of equations has been developed, from which the goniometer orientation may be determined [131].

## 7. Predicting the Future of Orientation Dependence: Outlook

The unparalleled precision with which NMR interaction parameters can be extracted from single-crystal data had people drawn to this method right from the early days of NMR spectroscopy [1,2,3,4,5]. With high probability, these advantages will prompt researchers to also make use of single-cystal NMR in the future. However, the high precision results available from measuring single crystals are often offset by the lack of availability of suitable systems, i.e., single crystals of sufficient size. One way to overcome this problem is to resort to naturally grown single crystals of minerals. This was also realised early on, as demonstrated by a ^19^F-NMR study of fluorite (CaF_2_) from 1946 [1], and ^9^Be- and ^27^Al-NMR measurements of beryl (Be_3_Al_2_Si_6_O_18_) from 1956 [5]. Another way to improve the signal-to-noise ratio is to put a limited number of small crystallites in a MAS rotor and spin it, as described in Section 3.2. While this approach certainly increases sensitivity, data acquisition and analysis differ very much from the strategies discussed in the current article. An approach that is fully compatible to established single-crystal methodology is the use of microcoils [132,133]. These coils allow for very small sample volume, while at the same time increasing the available RF power drastically, and may easily be combined with conventional goniometer probe designs. Finally, during recent years, dynamical nuclear polarisation (DNP) [134,135] has been rediscovered as a means to drastically increase the signal of the target nuclide. Successful application of this method has also been demonstrated for single-crystal samples [136,137,138,139].

Whatever strategies will be invented and adopted in the future for acquisition and analysis of NMR data from single crystals, it is very likely that the method as such will continue to be in use.

## Figures and Tables

**Table 1:** ^27^Al isotropic chemical shift values $\delta_{\mathrm{iso}}$ in the solid state for aluminium in various oxygen/nitrogen coordination environments.

^27^Al Coordination	$\delta_{\mathrm{iso}}$ Range ^a^	References
AlN_6_	−6 to 5 ppm	[40,41,42,43]
AlO_6_	−21 to 37 ppm	[44,45,46,47,48,49,50]
AlO_5_	20 to 52 ppm ^b^	[51,52,53,54,55,56]
AlO_4_	39 to 85 ppm	[46,47,48,49,50,57]
AlO_3_N_3_	43 to 55 ppm	[58]
AlO_*y*_N_4−*y*_	66 to 103 ppm	[59,60,61]
AlN_4_	100 to 115 ppm	[61,62,63,64,65,66]
Al_4_C_3_	111 to 120 ppm	[67]

^a^ Referenced against Al^3+^(aq). ^b^ Some of the resonances assigned to AlO_5_ may actually originate from strongly distorted AlO_4_ tetrahedra [54].

**Table 2:** Influence of the quadrupolar interaction (up to 3rd order), the chemical shift, and cross terms on the frequencies of the central transition (CT) and of the satellite transitions (ST) along with their combinations. Here, ✓ indicates *affected by*, ✗ indicates *not affected by*, and the arrows ↑↓ have the obvious meaning of *up* and *down*, respectively.

	1st Order	2nd Order	3rd Order	Chemical Shift	Cross Term
CT frequency	✗	✓	✗	✓	✓
ST frequencies	✓	✓	✓	✓	✓
Signs for ±k terms	≠	=	≠	=	=
ST difference (splittings)	✓	✗	✓	✗	✗
ST sum (centres)	✗	✓	✗	✓	✓
Scales with $\nu_{0}$	✗	↓	↓	↑	↓

**Table 3:** Chemical shift tensor components $\delta_{ii}$ and the resulting isotropic shift values $\delta_{\mathrm{iso}}$, as derived from single crystals under magic-angle spinning (MAS). The eigenvalues are ordered according to the Haeberlen convention (see Equation (7)), and for L-alanine, listed in the order C_*A*_ (carboxy), C_*B*_ (methylene), and C_*C*_ (methyl).

Nuclide	Compound	$\delta_{11}$	$\delta_{22}$	$\delta_{33}$	$\delta_{\mathrm{iso}}$	Ref.
(*spin I*)	(Formula)	(in ppm)				
^29^Si	Forsterite	−38.8	−55.3	−95.4	−63.2	[98]
(I=1/2)	(Mg_2_SiO_4_)					
^13^C (I=1/2)	L-alanine (C_3_H_7_NO_2_)	239.8	185.2	106.4	177.1	[100]
		65.4	54.0	31.3	50.2	
		31.2	19.5	8.4	19.7	

**Table 4:** The positions of the Pb and V atoms (in fractional coordinates) in the unit cell of vanadinite, Pb_5_(VO_4_)_3_Cl, which crystallises in space group $P6_{3}/m$ (No. 176), with Z=2 and lattice parameters a=b=10.299 Å, c=7.308 Å, as determined by X-ray diffraction analysis of a single crystal [110].

Atom	Wyckoff	Site	Atomic Coordinates		
	Position	Symmetry	x	y	z
Pb1	4f	3..	1/3	2/3	0.00786(5)
Pb2	6h	m..	0.25542(3)	0.01425(3)	1/4
V1	6h	m..	0.4097(2)	0.3836(1)	1/4

## Data Availability

Not applicable.

## Appendix A. Tensor Transformations

### Appendix A.1. The Euler Angles

Changing a coordinate system labeled XYZ into a new system xyz with different orientation, generally requires three rotations described by the angles α, β, and γ, about three different axes, as originally introduced by Leonhard Euler (1707–1783). This operation sequence also describes the transformation of tensors by rotation, see below. Obviously, there are several possible sequences to execute three successive rotations, so agreeing on a convention for the transformation XYZ→xyz is helpful. In the NMR community, the convention suggested by Morris E. Rose (1911–1967) is widely adopted [17]:

(A1) $$\begin{array}{ccc} \alpha & - & \mathrm{rotation}\;\mathrm{angle}\;\mathrm{about}\;\mathrm{the}\;\mathrm{original}\;Z-\mathrm{axis};\\ \beta & - & \mathrm{rotation}\;\mathrm{angle}\;\mathrm{about}\;\mathrm{the}\;\mathrm{new}\;Y^{\prime }-\mathrm{axis};\\ \gamma & - & \mathrm{rotation}\;\mathrm{angle}\;\mathrm{about}\;\mathrm{the}\;\mathrm{final}\;z-\mathrm{axis}. \end{array}$$

In Figure 1, it is shown how these consecutive rotations change the coordinate system in the sequence $XYZ\to X^{\prime }Y^{\prime }Z\to X^{\prime \prime }Y^{\prime }Z^{\prime \prime }\to xyz$.

For the description of NMR experiments, the task is often the transformation of an interaction tensor from a frame where it is well-defined, such as the principal axes system (PAS) or the crystal frame CRY, into the laboratory frame LAB, where the measurement takes place. In the LAB frame, the *z*-axis is defined by the field lines of the external magnetic field. Therefore, the third and final rotation around the *z*-axis by the angle γ is not necessary, as it would not change the outcome of the measurement, because the external magnetic field is cylindrically symmetric. Hence, the NMR frequencies of the various interactions (as itemised in Equation (1)) depend only on two angles, Ω=(α,β).

### Appendix A.2. Euler Rotation Matrices

The rotation matrices R realising the Euler rotations described above are defined by the following:

(A2) $$R\left(\alpha \right)=\left(\begin{array}{ccc} \cos\alpha & \sin\alpha & 0\\ -\sin\alpha & \cos\alpha & 0\\ 0 & 0 & 1 \end{array}\right)$$

(A3) $$R\left(\beta \right)=\left(\begin{array}{ccc} \cos\beta & 0 & -\sin\beta \\ 0 & 1 & 0\\ \sin\beta & 0 & \cos\beta \end{array}\right)$$

(A4) $$R\left(\gamma \right)=\left(\begin{array}{ccc} \cos\gamma & \sin\gamma & 0\\ -\sin\gamma & \cos\gamma & 0\\ 0 & 0 & 1 \end{array}\right).$$

For a full transformation involving all three angles, the rotation matrix R(α,β,γ) is the product of the individual matrices in the order of the applied rotations:

(A5) $$\begin{array}{ccc} R(\alpha ,\beta ,\gamma ) & = & R(\gamma )R(\beta )R(\alpha )\\ \; & = & \left(\begin{array}{ccc} \cos\alpha \cos\beta \cos\gamma -\sin\alpha \sin\gamma \; & \sin\alpha \cos\beta \cos\gamma +\cos\alpha \sin\gamma \; & -\sin\beta \cos\gamma \\ -\cos\alpha \cos\beta \sin\gamma -\sin\alpha \cos\gamma \; & -\sin\alpha \cos\beta \sin\gamma +\cos\alpha \cos\gamma \; & \sin\beta \sin\gamma \\ \cos\alpha \sin\beta \; & \sin\alpha \sin\beta \; & \cos\beta \end{array}\right). \end{array}$$

Conveniently, these rotation matrices are unitary, such that the inverse matrix is just the transpose of the original matrix, i.e., with rows and columns interchanged, $R^{-1}=R^{T}$.

### Appendix A.3. General Rotation Matrix

Rotation by an angle φ around an arbitrary axis $\vec{g}$, described in polar coordinates by $\vec{g}=\left(\begin{array}{ccc} g_{1} & g_{2} & g_{3} \end{array}\right)=\left(\begin{array}{ccc} \sin\theta \cos\phi & \sin\theta \sin\phi & \cos\theta \end{array}\right)$ is accomplished by the general rotation matrix K, as given by Granino and Theresa Korn [140]:

(A6) $$K=\cos\varphi \left(\begin{array}{ccc} 1 & 0 & 0\\ 0 & 1 & 0\\ 0 & 0 & 1 \end{array}\right)+\left(1-\cos\varphi \right)\left(\begin{array}{ccc} g_{1}^{2} & g_{1}g_{2} & g_{1}g_{3}\\ g_{2}g_{1} & g_{2}^{2} & g_{2}g_{3}\\ g_{3}g_{1} & g_{3}g_{2} & g_{3}^{2} \end{array}\right)+\sin\varphi \left(\begin{array}{ccc} 0 & -g_{3} & g_{2}\\ g_{3} & 0 & -g_{1}\\ -g_{2} & g_{1} & 0 \end{array}\right).$$

Using $\vec{g}=\left(\begin{array}{ccc} 1 & 0 & 0 \end{array}\right)$ as an example, the above expression indeed reduces to the standard Euler matrix for rotations around the *x*-axis:

(A7) $$R_{x}=\left(\begin{array}{ccc} 1 & 0 & 0\\ 0 & \cos\varphi & -\sin\varphi \\ 0 & \sin\varphi & \cos\varphi \end{array}\right).$$

### Appendix A.4. Active vs. Passive Rotations

A tensor transformation that rotates the coordinate system (i.e., expresses the tensor T in a new frame) is called a ‘passive’ rotation, whereas rotating a tensor in a fixed frame is called an ‘active’ rotation. Using the rotation matrices R defined above, the transformation

(A8) $$R^{-1}\left(\varphi \right)\cdot T\cdot R\left(\varphi \right)$$

performs an active rotation by the angle φ in the mathematically positive sense (i.e., counter-clockwise), and a passive rotation in the mathematically negative sense. A good description of this somewhat intricate issue may be found in References [22,141].

### Appendix A.5. Inversion and Reflection

The transformation matrix S for a mirror plane which has the vector $\vec{v}$ as its plane normal is described by the Householder transformation [142], and is given by ($\delta_{\mathrm{ij}}$ – Kronecker delta, $∥\vec{v}∥$ – norm of the vector):

(A9) $$S=\delta_{\mathrm{ij}}-2\frac{v_{i}v_{j}}{{∥\vec{v}∥}^{2}}=\frac{1}{{∥\vec{v}∥}^{2}}\left(\begin{array}{ccc} {∥\vec{v}∥}^{2}-2v_{1}^{2} & -2v_{1}v_{2} & -2v_{1}v_{3}\\ -2v_{2}v_{1} & {∥\vec{v}∥}^{2}-2v_{2}^{2} & -2v_{2}v_{3}\\ -2v_{3}v_{1} & -2v_{3}v_{2} & {∥\vec{v}∥}^{2}-2v_{3}^{2} \end{array}\right).$$

For the vector $\vec{c}=\left(\begin{array}{ccc} 0 & 0 & 1 \end{array}\right)$, this matrix reduces to the following:

(A10) $$S_{c}=\left(\begin{array}{ccc} 1 & 0 & 0\\ 0 & 1 & 0\\ 0 & 0 & -1 \end{array}\right).$$

## Appendix B. NMR Interaction Tensors from Single-Crystal Experiments

**Table A1:** ^207^Pb (I=1/2) chemical shift tensor components $\delta_{ii}$ in their principal axes system (PAS) and the resulting isotropic shift values $\delta_{\mathrm{iso}}$ ^a^, as determined by single-crystal NMR at room temperature. The eigenvalues are ordered according to the Haeberlen convention, see Equation (7).

Compound	Wyckoff	$\delta_{11}$	$\delta_{22}$	$\delta_{33}$	$\delta_{\mathrm{iso}}$	Ref.
(*Mineral*)	Position	(in ppm)				
PbCO_3_ (*cerussite*)	4c	−2315(1)	−2492(3)	−3071(3)	−2626(3)	[10]
Pb_5_(VO_4_)_3_Cl	4f	−1200(30)	−1250(31)	−2400(60)	−1620(40)	[124]
(*vanadinite*)	6h	−1000(5)	−1387(7)	−2800(14)	−1729(9)	
PbMoO_4_ (*wulfenite*)	4a	−2074(1)	−2074(1)	−1898(1)	−2015(1)	[129]
Pb_2_Cl_2_CO_3_ (*phosgenite*)	8k	−2553(1)	−1929(1)	−1301(1)	−1928(2)	[129]
PbSO_4_ (*anglesite*)	4c	−3365(3)	−3538(3)	−3942(4)	−3615(3)	[130]
Pb_5_(PO_4_)_3_Cl	4f	−2730(13)	−2780(13)	−2910(13)	−2810(14)	[143]
(*pyromorphite*)	6h	−1771(9)	−1826(9)	−2920(13)	−2170(11)	

^a^ All chemical shift values were referenced indirectly to ^1^H in 100% TMS at −0.1240 ppm, which is equivalent to the common Pb(NO_3_)_2_-powder referencing at $\delta_{iso}=-3492$ ppm.

**Table A2:** Quadrupolar coupling constants χ (Equation (9)) and asymmetry parameters $\eta_{Q}$ (Equation (11)) of various nuclides with half-integer spin I>1/2 in periodic structures, plus their isotropic shift values $\delta_{\mathrm{iso}}$, as derived from single-crystal NMR measurements. Multiple entries for one compound are for multiple sites in the crystal structure.

Nuclide	Spin *I*	Compound	χ	$\eta_{Q}$	$\delta_{\mathrm{iso}}$	Ref.
		(*Mineral*)	(in MHz)		(in ppm)	
^7^Li	3/2	LiH_2_PO_4_	0.071(1)	0.37(2)	−0.2(1) ^a^	[144]
		γ-LiAlO_2_	0.1151(6)	0.69(1)	82 ^a^	[145,146]
^9^Be	3/2	Be_3_Al_2_Si_6_O_18_	0.504(4)	0.090(5)	−1.9(1) ^a^	[5,147]
		(*beryl*)				
^11^B	3/2	CaB_3_O_4_ (OH)_3_· H_2_O	0.312(1)	0.809(3)	1.2(2)	[126]
		(*colemanite*)	2.543(5)	0.055(4)	17.1(3)	
		Na_2_B_4_O_7_· 10H_2_O	0.497(1)	0.624(3)	2.1(1)	
		(*borax*)	2.565(3)	0.105(2)	18.2(1)	
^69^Ga	3/2	CdGa_2_Te_4_	4.18	0	120	[148]
			5.91	0	290	
^71^Ga	3/2	Y_3_Ga_5_O_12_	4.10(6)	0.03(4)	6(1)	[149]
			13.1(2)	0.05(3)	219(19)	
^87^Rb	3/2	RbClO_4_	3.30(4)	0.21(3)	−13.7(6)	[150]
		Rb_2_SO_4_	2.72(3)	0.930(3)	42.6(3)	
			5.29(5)	0.12(3)	16(1)	[150]
		Rb_2_CrO_4_	9.43(6)	0.70(1)	−60(3)	
			3.55(1)	0.36(1)	−8.7(2)	[151]
		RbVO_3_	9.69(1)	0.748(6)	−36(1)	[152]
^27^Al	5/2	AlN	1.914(1)	0	113.6(3)	[81]
		α-Al_2_O_3_	2.40(1)	0.01(9)	18.8(3)	[153]
		γ-LiAlO_2_	3.330(5)	0.656(2)	81.8(3)	[127]
		Al_2_SiO_5_	5.8323(8)	0.6733(2)	35.3(5)	[154]
		(*andalusite*)	15.261(7)	0.1029(3)	9.7(5)	
^67^Zn	5/2	Zn(CH_3_COO)_2_ · 2H_2_O	5.34(3)	0.82(2)	−123(3)	[155]
		Zn(HCOO)_2_ · 2H_2_O	6.340(5)	0.98(1)	−24(7)	[156]
			9.63(6)	0.45(2)	−26(9)	
^45^Sc	7/2	[{Sc(H_2_O)_5_(μ-OH)}_2_]Cl_4_·2H_2_O	14.613(6)	0.5409(4)	18.8(9)	[157]
^51^V	7/2	V_2_O_5_	0.805(6)	0.04(1)	−612 ^a^	[125,158]
		Pb_5_(VO_4_)_3_Cl	2.520(1)	0.047(3)	−509(3)	[124]
^133^Cs	7/2	Cs_2_CrO_4_	0.138(6)	0.15(1)	27(3)	[159]
			0.38(1)	0.52(2)	−100(2)	

^a^ Isotropic chemical shift from MAS-NMR of a polycrystalline sample.

**Table A3:** Quadrupolar coupling constants χ and asymmetry parameters $\eta_{Q}$ of deuterium (^2^H, spin I=1) in aromatic moieties of organic compounds, as derived from single-crystal NMR measurements.

Compound		Deuteron(s) ^a^	χ (in kHz)	$\eta_{Q}$	Ref.
Name	Formula				
Benzene	C_6_D_6_	all	187 ^b^	0 ^b^	[160]
Pyromellitic acid	C_6_D_2_(COOD)_4_	D_3_, D_3′_	168	0.075	[161]
Azulene	C_10_H_6_D_2_	D_1_, D_3_	182	0.06	[162]
2,3-Dimethyl-naphthalene	C_10_H_6_(CH_3_)_2_	D_1,2,3_	177	0	[163]
Fluorene	C_13_D_10_	D_1,4_	178.7	0.047	[136]
		D_5,8_	177.3	0.040	
		D_2_	179.9	0.047	
		D_3_	180.0	0.047	
Anthracene	C_14_D_10_	(B, F, D)	179.0	0.058	[164]
		A	183.7	0.067	
		G	181.3	0.055	

^a^ The atomic labels (often designating the attached carbon atoms rather than the deuterons) are those used in the original reference. Listing of several deuterons for one χ, $\eta_{Q}$ pair indicates crystallographic equivalence, i.e., the deuteron positions are related by a symmetry element of the crystal structure. ^b^ Values derived from motionally averaged tensor, assuming $\eta_{Q}=0$.

## Appendix C. Coefficients for Quadrupole Interaction 2nd and 3rd Order

As described above in Section 2.2, the quadrupole interaction term may be considered as a minor perturbation to the energy levels derived from the Zeman interaction, as long as the quadrupolar coupling constant χ is much smaller than the Larmor frequency. The expressions for the various perturbation orders for this ‘high-field case’ can be found in the literature in many places, see for example [2,4,68,165,166,167,168,169]. Whereas the first-order shift of the satellites was derived and published as early a 1950 by Robert V. Pound (1919–2010) [2], the results for second order are in most instances given only for the central transition and not for the satellites. In addition, the original articles are often beset by typographical errors. To the best of our knowledge, the following expressions are correct. They are re-written from the terms given by Wolf et al. in 1970 [168].

The coefficients for the functions *g* and *f* of Equation (20) (with η being the short notation for $\eta_{Q}$) are as follows:

$$\begin{array}{cc} A^{\left(2\right)}=-\frac{27}{8}+\frac{9}{4}\eta \cos2\alpha -\frac{3}{8}\eta^{2}\cos^{2}2\alpha \; & \;D^{\left(2\right)}=-\frac{3}{2}+\eta \cos2\alpha -\frac{1}{6}\eta^{2}\cos^{2}2\alpha \\ B^{\left(2\right)}=\frac{30}{8}-\frac{1}{2}\eta^{2}-2\eta \cos2\alpha +\frac{3}{4}\eta^{2}\cos^{2}2\alpha \; & \;E^{\left(2\right)}=\frac{3}{2}-\frac{1}{6}\eta^{2}-\eta \cos2\alpha +\frac{1}{3}\eta^{2}\cos^{2}2\alpha \\ C^{\left(2\right)}=-\frac{3}{8}+\frac{1}{3}\eta^{2}-\frac{1}{4}\eta \cos2\alpha -\frac{3}{8}\eta^{2}\cos^{2}2\alpha \; & \;F^{\left(2\right)}=\frac{1}{6}\eta^{2}-\frac{1}{6}\eta^{2}\cos^{2}2\alpha \end{array}$$

The coefficients for the functions *u*, *v* and *w* of Equation (22) are given by the following:

$$\begin{array}{c} \begin{array}{cc} A^{\left(3\right)} & =-\frac{9}{4}+\frac{9}{4}\eta \cos2\alpha -\frac{3}{4}\eta^{2}\cos^{2}2\alpha +\frac{1}{12}\eta^{3}\cos^{3}2\alpha \\ B^{\left(3\right)} & =3-\frac{1}{4}\eta^{2}-\frac{7}{2}\eta \cos2\alpha +\frac{1}{12}\eta^{3}\cos2\alpha +\frac{19}{12}\eta^{2}\cos^{2}2\alpha -\frac{1}{4}\eta^{3}\cos^{3}2\alpha \\ C^{\left(3\right)} & =-\frac{3}{4}+\frac{1}{3}\eta^{2}+\frac{5}{4}\eta \cos2\alpha -\frac{1}{6}\eta^{3}\cos2\alpha -\frac{11}{12}\eta^{2}\cos^{2}2\alpha +\frac{1}{4}\eta^{3}\cos^{3}2\alpha \\ D^{\left(3\right)} & =-\frac{1}{12}\eta^{2}+\frac{1}{12}\eta^{3}\cos2\alpha +\frac{1}{12}\eta^{2}\cos^{2}2\alpha -\frac{1}{12}\eta^{3}\cos^{3}2\alpha \\ E^{\left(3\right)} & =\frac{9}{16}-\frac{9}{16}\eta \cos2\alpha +\frac{3}{16}\eta^{2}\cos^{2}2\alpha -\frac{1}{48}\eta^{3}\cos^{3}2\alpha \\ F^{\left(3\right)} & =-\frac{21}{16}+\frac{1}{4}\eta^{2}+\frac{11}{16}\eta \cos2\alpha -\frac{1}{12}\eta^{3}\cos2\alpha -\frac{13}{48}\eta^{2}\cos^{2}2\alpha +\frac{1}{16}\eta^{3}\cos^{3}2\alpha \\ G^{\left(3\right)} & =\frac{15}{16}-\frac{1}{12}\eta^{2}-\frac{3}{16}\eta \cos2\alpha +\frac{1}{12}\eta^{3}\cos2\alpha -\frac{1}{48}\eta^{2}\cos^{2}2\alpha -\frac{1}{16}\eta^{3}\cos^{3}2\alpha \\ H^{\left(3\right)} & =-\frac{3}{16}+\frac{1}{16}\eta \cos2\alpha +\frac{5}{48}\eta^{2}\cos^{2}2\alpha +\frac{1}{48}\eta^{3}\cos^{3}2\alpha \\ I^{\left(3\right)} & =\frac{3}{4}-\frac{3}{4}\eta \cos2\alpha +\frac{1}{4}\eta^{2}\cos^{2}2\alpha -\frac{1}{36}\eta^{3}\cos^{3}2\alpha \\ J^{\left(3\right)} & =-\frac{3}{2}+\frac{1}{4}\eta^{2}+\eta \cos2\alpha -\frac{1}{12}\eta^{3}\cos2\alpha -\frac{5}{12}\eta^{2}\cos^{2}2\alpha +\frac{1}{12}\eta^{3}\cos^{3}2\alpha \\ K^{\left(3\right)} & =\frac{3}{4}-\frac{1}{6}\eta^{2}-\frac{1}{4}\eta \cos2\alpha +\frac{1}{9}\eta^{3}\cos2\alpha +\frac{1}{12}\eta^{2}\cos^{2}2\alpha -\frac{1}{12}\eta^{3}\cos^{3}2\alpha \\ L^{\left(3\right)} & =-\frac{1}{12}\eta^{2}-\frac{1}{36}\eta^{3}\cos2\alpha +\frac{1}{12}\eta^{2}\cos^{2}2\alpha +\frac{1}{36}\eta^{3}\cos^{3}2\alpha \end{array} \end{array}$$

## Appendix D. Equivalence of Tensor and Polar/Azimuthal Angle Notation

Using the chemical shift tensor δ as an example, it will be shown here that the frequency of the chemical shift $\nu_{\mathrm{CS}}$ calculated from Equation (3) is identical to that derived from the vector-tensor-vector product (Equation (32)) introduced in Section 4. This can be done comparatively quickly by using the principal axes system (PAS) as a common coordinate system for both tensor and vector (other coordinate systems may be used as well, but involve additional transformations). Thus, the relevant tensor is $\delta^{\mathrm{PAS}}$, which has its eigenvalues on the diagonal, and is usually given in ppm. Adapting Equation (32), the chemical shift frequency may be calculated from the following:

(A11) $$\frac{\nu_{\mathrm{CS}}}{\nu_{0}}={\vec{b}}^{T}\cdot \left(\begin{array}{ccc} \delta_{11} & 0 & 0\\ 0 & \delta_{22} & 0\\ 0 & 0 & \delta_{33} \end{array}\right)\cdot \vec{b}$$

Using the symbol β for the polar, and α for the azimuthal angle of the spherical coordinates in the PAS frame, the vector of the magnetic field (see also Equation (31)) is given by the following:

$$\vec{b}=\left(\begin{array}{c} \sin\beta \cos\alpha \\ \sin\beta \sin\alpha \\ \cos\beta \end{array}\right).$$

Executing the product of Equation (A11) leads to the following:

(A12) $$\begin{array}{ccc} \frac{\nu_{\mathrm{CS}}}{\nu_{0}} & = & \delta_{11}\sin^{2}\beta \cos^{2}\alpha +\delta_{22}\sin^{2}\beta \sin^{2}\alpha +\delta_{33}\cos^{2}\beta \\ \; & = & \left[\delta_{11}\cos^{2}\alpha +\delta_{22}\sin^{2}\alpha \right]\sin^{2}\beta +\delta_{33}\cos^{2}\beta \end{array}.$$

To reorganise the above expression to the desired form, the auxiliary parameters *C* and ϵ are introduced:

(A13) $$\begin{array}{cc} \delta_{11}=C+\epsilon & \delta_{22}=C-\epsilon \end{array}.$$

Therefore, the following is true:

(A14) $$\epsilon =\frac{1}{2}\left(\delta_{11}-\delta_{22}\right).$$

Using a rearranged form of the definition of the isotropic chemical shift (Equation (5)) and substituting $\delta_{11}$ and $\delta_{22}$ according to Equation (A13), we obtain the following:

$$3\delta_{\mathrm{iso}}=\delta_{11}+\delta_{22}+\delta_{33}=C+\epsilon +C-\epsilon +\delta_{33}.$$

Thus,

(A15) $$C=\frac{1}{2}\left(3\delta_{\mathrm{iso}}-\delta_{33}\right).$$

With the help of the trigonometric relationships,

$$\begin{array}{ccc} \cos^{2}x+\sin^{2}x & = & 1\\ \cos^{2}x-\sin^{2}x & = & \cos2x \end{array}.$$

Equation (A12) can now be rearranged as follows:

(A16) $$\begin{array}{ccc} \frac{\nu_{CS}}{\nu_{0}}\; & = & \left[\left(C+\epsilon \right)\cos^{2}\alpha +\left(C-\epsilon \right)\sin^{2}\alpha \right]\sin^{2}\beta +\delta_{33}\cos^{2}\beta \\ \; & = & \left[C\left(\cos^{2}\alpha +\sin^{2}\alpha \right)+\epsilon \left(\cos^{2}\alpha -\sin^{2}\alpha \right)\right]\sin^{2}\beta +\delta_{33}\cos^{2}\beta \\ \; & = & C\sin^{2}\beta +\epsilon \cos2\alpha \sin^{2}\beta +\delta_{33}\cos^{2}\beta \end{array}.$$

Substituting parameters ϵ and *C* (Equations (A14) and (A15)), we obtain the following:

(A17) $$\begin{array}{ccc} \frac{\nu_{\mathrm{CS}}}{\nu_{0}} & = & C\sin^{2}\beta +\epsilon \cos2\alpha \sin^{2}\beta +\delta_{33}\cos^{2}\beta \\ \; & = & \frac{1}{2}\left(3\delta_{\mathrm{iso}}-\delta_{33}\right)\sin^{2}\beta +\delta_{33}\cos^{2}\beta +\epsilon \cos2\alpha \sin^{2}\beta \\ \; & = & \frac{1}{2}\left[\left(3\delta_{\mathrm{iso}}-\delta_{33}\right)\left(1-\cos^{2}\beta \right)+2\delta_{33}\cos^{2}\beta +2\epsilon \cos2\alpha \sin^{2}\beta \right]\\ \; & = & \frac{1}{2}\left[3\delta_{\mathrm{iso}}-\delta_{33}+3\left(\delta_{33}-\delta_{\mathrm{iso}}\right)\cos^{2}\beta +\left(\delta_{11}-\delta_{22}\right)\cos2\alpha \sin^{2}\beta \right]\\ \; & = & \delta_{\mathrm{iso}}+\frac{1}{2}\left[\delta_{\mathrm{iso}}-\delta_{33}+3\left(\delta_{33}-\delta_{\mathrm{iso}}\right)\cos^{2}\beta -\left(\delta_{22}-\delta_{11}\right)\cos2\alpha \sin^{2}\beta \right] \end{array}.$$

To proceed, we repeat the definitions of Equation (6):

$$\eta_{\mathrm{CS}}=\frac{\delta_{22}-\delta_{11}}{\Delta \delta }\;\;\Delta \delta =\delta_{33}-\delta_{iso}.$$

The asymmetry parameter $\eta_{CS}$ and the reduced anisotropy Δδ can now be introduced into Equation (A17) to write an expression. which is equivalent to Equation (3) (except for $\nu_{0}$ still being on the left-hand-side):

(A18) $$\begin{array}{ccc} \frac{\nu_{\mathrm{CS}}}{\nu_{0}} & = & \delta_{\mathrm{iso}}+\frac{1}{2}\left[-\Delta \delta +3\Delta \delta \cos^{2}\beta -\left(\delta_{22}-\delta_{11}\right)\cos2\alpha \sin^{2}\beta \right]\\ \; & = & \delta_{\mathrm{iso}}+\frac{\Delta \delta }{2}\left[3\cos^{2}\beta -1-\eta_{\mathrm{CS}}\cos2\alpha \sin^{2}\beta \right] \end{array}.$$
